# An ensemble of B-DNA dinucleotide geometries lead to characteristic nucleosomal DNA structure and provide plasticity required for gene expression

**DOI:** 10.1186/1472-6807-11-1

**Published:** 2011-01-05

**Authors:** Arvind Marathe, Manju Bansal

**Affiliations:** 1 Molecular Biophysics Unit, Indian Institute of Science, Bangalore - 12, India

## Abstract

**Background:**

A nucleosome is the fundamental repeating unit of the eukaryotic chromosome. It has been shown that the positioning of a majority of nucleosomes is primarily controlled by factors other than the intrinsic preference of the DNA sequence. One of the key questions in this context is the role, if any, that can be played by the variability of nucleosomal DNA structure.

**Results:**

In this study, we have addressed this question by analysing the variability at the dinucleotide and trinucleotide as well as longer length scales in a dataset of nucleosome X-ray crystal structures. We observe that the nucleosome structure displays remarkable local level structural versatility within the B-DNA family. The nucleosomal DNA also incorporates a large number of kinks.

**Conclusions:**

Based on our results, we propose that the local and global level versatility of B-DNA structure may be a significant factor modulating the formation of nucleosomes in the vicinity of high-plasticity genes, and in varying the probability of binding by regulatory proteins. Hence, these factors should be incorporated in the prediction algorithms and there may not be a unique 'template' for predicting putative nucleosome sequences. In addition, the multimodal distribution of dinucleotide parameters for some steps and the presence of a large number of kinks in the nucleosomal DNA structure indicate that the linear elastic model, used by several algorithms to predict the energetic cost of nucleosome formation, may lead to incorrect results.

## Background

The nucleosome is the fundamental repeating unit of the eukaryotic chromosome [1,2]. Nucleosome positioning is controlled by several factors such as intrinsic preference of the DNA sequence to assume a nucleosome-like structure, higher order chromatin organisation, DNA methylation and presence of DNA binding proteins such as transcription factors [3]. A large number of studies have tried to characterise the intrinsic preferences of the DNA sequence and derive the complete sequence pattern of nucleosomal DNA by analysing the in-phase and out-of-phase occurrences of various dinucleotides such as AA and TT, GG and CC, AT, TA, and CA and TG [4-11]. However, formation of a large number of nucleosomes is primarily controlled by factors other than the nucleosome sequence [12-14]. In this context, one needs to ask what role, if any, is played by the structural variability of nucleosomal DNA in the formation of these nucleosomes.

The high resolution crystal structures of the nucleosome core particle [15] comprise of a left-handed superhelix covering ~1.7 turns, with a pseudo two-fold axis of symmetry passing through one of the central basepairs, where the major groove faces the histone octamer. Since the interactions of the histone core with DNA are primarily non-specific, the ability of the DNA sequence to assume this structure determines the stability of the nucleosome core. The nucleosome structure at the local level is described in terms of dinucleotide step parameters tilt, roll, twist, shift, slide and rise that quantify the motion between adjacent basepair planes [16]. Studies of the best resolved nucleosome structure (PDB id: 1KX5, [15]) have shown that the parameter roll is primarily responsible for curvature of the nucleosome [17], while twist and slide contribute predominantly to the pitch of the nucleosome [17-19]. Analysis of data from nucleosome crystal structures along with that from molecular dynamics simulations of the nucleosome structure have indicated that the distributions of roll, twist and slide are conserved, while tilt, shift and rise are relatively free [19].

In the present study, we have analysed the variability at the dinucleotide as well as longer length scales in a dataset of nucleosome X-ray crystal structures. Our analysis shows that even for identical sequences, there is significant local level structural variation and some amount of variation at longer length scales, indicating that there is a thermodynamic ensemble of local level structures that can give rise to the core nucleosome structure. These results also raise questions about the rationale for using only the best resolved X-ray crystal structure (PDB id: 1KX5, [15]) of nucleosome as the prototype on which different potential nucleosome sequences are threaded, for calculating the energetic cost of nucleosome formation [18,20-22]. Further, they indicate that use of the simple harmonic approximation to calculate the energetic cost of nucleosome formation [18,20-24] may lead to incorrect results, owing to the multimodal distribution of dinucleotide parameters for some steps [25,26] and the presence of a large number of kinks in the nucleosome structure.

## Results

The structural parameters of the nucleosome dataset were analysed to gain a perspective on the intrinsic variability of the nucleosomal DNA structure. The dataset comprised of twenty-nine structures corresponding to only six unique sequences (see 'Methods' section for details). However, even for structures with identical sequences, the local structural parameters were observed to vary considerably. Hence all the structures have been retained for the analysis.

Table 1 shows the distribution of the ten unique dinucleotide steps for all the six sequences. The last column in Table 1 gives the total number and proportion of each dinucleotide step in the entire dataset of twenty-nine crystal structures. It is seen from Table 1 that all the sequences have large proportions of AA/TT and CA/TG (> 15% for five sequences) dinucleotide steps. There are also significant proportions of AG/CT, GA/TC and AT/AT steps (> 10% for sequences 2-5). Compared to other sequences, sequence 1 has a larger proportion of AA/TT and GA/TC steps, and smaller proportions of CA/TG and AT/AT steps. The proportion of CG/CG and TA/TA steps is very small for all the sequences.

**Table 1:** Occurrence of the ten dinucleotide steps for each sequence in each of the six nucleosomal DNA sequences

	Occurrence						
Step	Sequence number						Total
	1	2	3	4	5	6	
AA/TT	32 (22.1)	25 (17.4)	27 (18.8)	28 (19.3)	28 (19.3)	25 (18.1)	805 (19.1)

AG/CT	18 (12.4)	18 (12.5)	16 (11.1)	18 (12.4)	16 (11.0)	18 (12.3)	478 (11.4)

GA/TC	22 (15.2)	16 (11.1)	18 (12.5)	18 (12.4)	18 (12.4)	16 (11.0)	520 (12.4)

GG/CC	12 (8.3)	12 (8.3)	14 (9.7)	12 (8.3)	14 (9.7)	14 (9.6)	396 (9.4)

AC/GT	14 (9.7)	14 (9.7)	12 (8.3)	12 (8.3)	12 (8.3)	14 (9.6)	356 (8.5)

AT/AT	11 (7.6)	15 (10.4)	15 (10.4)	15 (10.3)	15 (10.3)	15 (10.3)	431 (10.3)

GC/GC	6 (4.1)	8 (5.6)	8 (5.6)	8 (5.5)	8 (5.5)	8 (5.5)	230 (5.5)

CA/TG	18 (12.4)	28 (19.4)	26 (18.1)	28 (19.3)	26 (17.9)	28 (19.2)	758 (18.0)

CG/CG	6 (4.1)	0 (0.0)	2 (1.4)	0 (0.0)	2 (1.4)	0 (0.0)	50 (1.2)

TA/TA	6 (4.1)	8 (5.6)	6 (4.2)	6 (4.1)	6 (4.1)	6 (4.1)	180 (4.3)

Overall	145	144	144	145	145	146	4204

The last column lists the total size of the dataset for the entire dataset of twenty-nine crystal structures. Thus if N*_i _*is the occurrence for a step in sequence i, then the number in the last column is calculated as N_1_+N_2_+2*N_3_+3*N_4_+20*N_5_+2*N_6_. The numbers in parentheses denote the population as percentages, of the total size for the respective sequence, and each column adds up to 100.

Of the total of 4204 steps, 4198 steps incorporate Watson-Crick basepairs. The remaining 6 steps are part of three TCA/TGA trinucleotides and each incorporates a non-Watson-Crick C:G basepair. All the 58 terminal steps are AT/AT, and incorporate Watson-Crick basepairs. Only the 4140 non-terminal steps incorporating Watson-Crick basepairs have been considered for all the analysis described in this work.

A detailed analysis of all the protein-DNA contacts for all the structures was carried out. Most of the specific protein contacts with DNA base atoms as well as non-specific contacts with the DNA backbone were conserved in terms of distance from SHL 0. Exceptions were observed in a few cases which were the result of mutations in the histones. However, the pattern of variation in structural paramaters observed in this study seems to be independent of protein contacts, and more a result of position dependence and the intrinsic properties of the dinucleotide steps. Hence interactions of DNA with the protein have not been focussed upon in this study.

An analysis of all the inter-particle contacts in the crystal lattice was also carried out for all the structures. The only noteworthy observation is the loose twisting around SHL ±2 and tighter twisting around ±5, of the structure 2NZD of the human NCP, as compared to the NCP structures of Xenopus laevis, and also those of other organisms. This can be attributed to the DNA-DNA contacts in the human NCP, as commented by Tsunaka et al. [27]. Apart from this variation, none of the observed differences between the nucleosomal DNA structures can be related to differences in the inter-particle contacts in the crystal lattice. Hence the crystal packing effects will not be discussed further.

### Position-specific variation in the structure of the backbone, and of the dinucleotide steps

The backbone torsion angles *α*, *β*, *γ*, *δ *and *χ *do not show any significant variation from the standard B-DNA values. The major backbone flexibility is observed in the occurrence of BI and BII conformations.

Table 2 shows the overall proportions of the backbone torsion angle states as defined in the 'Methods' section. 68.3% of the nucleotides are found to take up state 1, with canonical values for *α*, *γ *and *ϵ*-*ζ *, while 20.4% of the nucleotides are observed to assume state 7 with canonical values for *α *and *γ *but with the BII conformation for *ϵ*-*ζ *. The remaining ~10% of the nucleotides are divided between the rest of the states, with state 6 occurring in ~5% of them.

**Table 2:** Occurence of the seven states for the backbone conformations, defined according to [63]

	Description			
State	*α*(°)	*γ*(°)	*ϵ*-*ζ *(°)	Occurrence
1	150-360	0-125 or 270-360	BI	5657 (68.3)
2	220-360	125-270	-	77 (0.9)
3	0-220	125-240	BII	155 (1.9)
4	0-150	0-125	-	39 (0.5)
5	0-220	125-240	BI	225 (2.7)
6	0-220	240-270	-	439 (5.3)
7	150-360	0-125 or 270-360	BII	1688 (20.4)

Numbers in parentheses indicate percentage occurrence.

A comparison with the distribution of backbone torsion angles for the free and protein-bound DNA reported by Marathe et al. [25] shows that the occurrence of the classical state 1 is lower than that observed in free oligomers or either of the protein-bound datasets. On the other hand, the proportion of state 7 is larger for the nucleosome dataset as compared to any of the datasets reported by Marathe et al., and is closest to the proportion for the free B-DNA dataset. It must be noted here that the 'Complex' dataset analysed by Marathe et al. also included two representative nucleosome structures 1KX5 and 1KX3. When these two structures are excluded from the 'Complex' dataset, the proportion of state 1 increases from 78.8% to 80.5% and the proportion of state 7 decreases from 10.0% to 7.6%. Thus DNA in the nucleosome structure has lower preference for the canonical backbone conformation as compared to protein-free and other protein-bound DNA, and the proportion of state 7 among the non-canonical conformations is larger than the corresponding proportions in protein-free and other protein-bound DNA structures.

**Figure 1:**
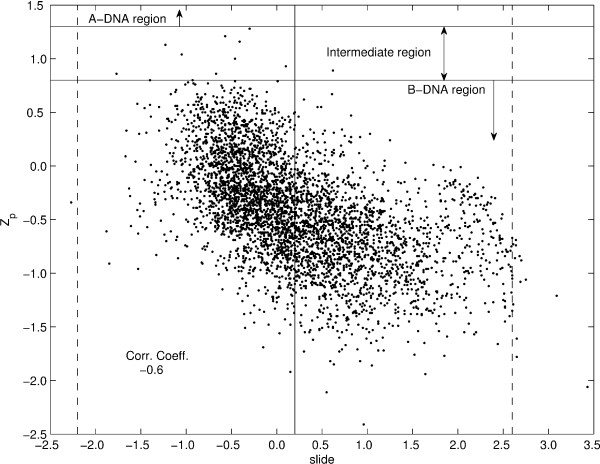
**Z*_p _*versus slide**. Z*_p _*versus slide for the nucleosomal DNA dataset. The two solid horizontal lines at Z*_p _*= 1.3 Å and Z*_p _*= 0.8 Å denote the cutoff values for classifying a dinucleotide step as A or B-DNA like, with Z*_p _*> 1.3 Å indicating an A-like structure, Z*_p _*≤ 0.8 Å indicating a B-like structure, and 0.8 Å < Z*_p _*≤ 1.3 Å indicating an intermediate structure. The solid vertical line at slide = 0.2 Å denotes the mean value for slide in free B-DNA, while the two dashed vertical lines at slide = -2.2 Å and 2.6 Å denote the values 3*σ *away from the free B-DNA mean value for slide. All the indicated values are based on the analysis of high resolution X-ray crystal structures of DNA [25].

**Figure 2:**
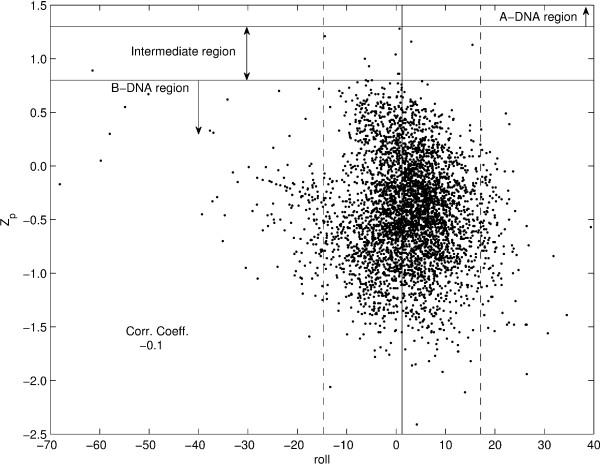
**Z*_p _*versus roll**. Z*_p _*versus roll for the nucleosomal DNA dataset. The solid vertical line at roll = 1.2° denotes the mean value for roll in free B-DNA, while the two dashed vertical lines at roll = -14.7° and 17.1° denote the values 3*σ *away from the free B-DNA mean value for roll. The rest of the specifications are as described in the caption for figure 1.

In case of dinucleotide step parameters, an important question is whether the nucleosomal DNA is primarily A-like or B-like at the local structural level. It has been shown earlier [25,28] that the parameter Z*_p _*best indicates whether a given dinucleotide step has an A-like or B-like structure. A value of Z*_p _*> 1.3 Å is considered an A-like conformation while Z*_p _*≤ 0.8 Å is considered as B-like conformation, with values in between the two cutoffs signifying an intermediate conformation [25]. A survey of the Z*_p _*values for all the dinucleotide steps in this study indicates that only 10 out of 4146 steps have a Z*_p _*value > 0.8 Å, indicating that almost the entire nucleosome dataset is B-DNA like. Of these, only one step in the structure 1KX4 has an A-like Z*_p _*value of 1.3 Å, while the other nine steps have Z*_p _*values signifying an intermediate conformation. The plots of Z*_p _*versus slide and Z*_p _*versus roll for the nucleosomal DNA dataset, shown in Figure 1 and 2 respectively, indicate that slide is correlated with Z*_p_*, while roll shows no correlation with Z*_p_*. Thus the observation in case of high-resolution free and protein-bound DNA crystal structures that slide, in addition to Z*_p_*, can discriminate between A and B-forms, while roll does not discriminate between the two forms [25], is also valid in case of nucleosomal DNA.

The rotational and translational motion between Watson-Crick basepairs constituting a dinucleotide step is observed to vary depending on their orientation with respect to the histone octamer and on the backbone conformation. The most significant variations are observed in case of the rotational parameters roll and twist, and the translational parameter slide, and the mean and standard deviation values for these parameters are shown in Tables 3, 4 and 5 respectively, for the ten Watson-Crick dinucleotide steps. The corrresponding values for the rotational parameter tilt, and the translational parameters shift and rise, are reported in tables S1, S2 and S3 of additional file 1.

**Table 3:** Mean and standard deviation values (given in parentheses, reported only for datasets with size ≥ 5) for roll, for the ten dinucleotide steps, incorporating Watson-Crick basepairs

	Roll values for dinucleotide steps with								

	Minor groove facing the histone octamer			Backbone facing the histone octamer			Major groove facing the histone octamer		
Step	(Region I)			(Region II)			(Region III)		
	BI/BI	BI/BII	BII/BII	BI/BI	BI/BII	BII/BII	BI/BI	BI/BII	BII/BII
AA/TT	-0.6 (5.3)	-6.5 (6.6)	-11.8 (7.6)	2.6 (4.7)	-2.5 (4.6)	-14.9 NA	7.0 (5.1)	4.8 (5.2)	6.6 NA
	241	67	8	122	21	1	289	55	1

AG/CT	5.4 (4.9)	-3.6 (4.6)	-6.5 (4.9)	5.2 (5.7)	19.9 NA	NA	10.1 (5.5)	8.5 (4.1)	NA
	104	119	47	29	2	0	139	38	0

GA/TC	1.1 (3.7)	-2.7 (5.7)	0.9 NA	7.1 (5.0)	6.6 (4.2)	8.8 NA	5.0 (4.4)	4.3 (3.7)	-4.3 NA
	82	117	2	73	31	3	153	54	2

GG/CC	4.7 (4.3)	-2.5 (9.7)	-21.7 (20.8)	7.2 (6.3)	5.9 (4.4)	3.0 NA	11.4 (6.4)	11.0 (5.8)	NA
	71	150	28	42	16	1	75	13	0

AC/GT	-6.5 NA	-5.8 NA	NA	4.3 (4.0)	15.1 NA	NA	4.1 (4.4)	1.9 (6.0)	NA
	1	4	0	59	1	0	271	20	0

AT/AT	-3.4 (3.4)	-3.5 (8.5)	NA	1.0 (3.5)	-5.5 NA	NA	2.5 (5.8)	0.0 (4.7)	NA
	109	5	0	110	4	0	139	6	0

GC/GC	0.3 (7.3)	-4.8 (5.0)	-8.0 (3.6)	6.3 (4.4)	0.6 (3.4)	NA	3.6 (3.0)	4.2 NA	NA
	32	102	12	29	28	0	26	1	0

CA/TG	6.6 (3.7)	-1.2 (8.6)	-13.0 (8.4)	7.3 (5.7)	4.0 (5.1)	-9.9 (6.2)	12.2 (6.9)	6.6 (6.4)	-6.1 (6.0)
	54	65	120	72	82	54	158	99	51

CG/CG	6.0 NA	-2.7 (3.8)	-4.8 (4.1)	NA	NA	NA	13.7 NA	-3.2 NA	NA
	3	35	10	0	0	0	1	1	0

TA/TA	-1.1 (6.7)	-3.7 NA	-36.2 NA	-2.0 (5.4)	-4.7 (4.4)	-4.3 NA	8.1 (6.6)	12.5 (7.0)	6.3 NA
	33	2	1	15	16	1	82	29	1

Overall	1.1 (5.8)	-3.4 (7.0)	-11.9 (11.1)	4.2 (5.5)	2.7 (6.2)	-8.8 (7.4)	6.9 (6.3)	6.4 (6.2)	-5.6 (6.3)
	730	666	228	551	201	60	1333	316	55

For each dinucleotide step, the 3*^rd ^*row lists the size of the population for the respective dataset. Based on the side of the DNA facing the histone octamer (minor groove, backbone or major groove), the dinucleotide steps have been divided into three groups. The population in each group has been further sub-divided depending on whether the backbone conformation was BI in both strands, or a mixture of BI and BII, or BII in both strands. Please refer 'Methods' section for details of the classification scheme.

**Table 4:** Mean and standard deviation values (given in parentheses, reported only for datasets with size ≥ 5) for twist, for the ten dinucleotide steps, i ncorporating Watson-Crick basepairs

	Twist values for dinucleotide steps with								

	Minor groove facing the histone octamer			Backbone facing the histone octamer			Major groove facing the histone octamer		
Step	(Region I)			(Region II)			(Region III)		
	BI/BI	BI/BII	BII/BII	BI/BI	BI/BII	BII/BII	BI/BI	BI/BII	BII/BII
AA/TT	34.3 (3.7)	38.7 (3.8)	41.2 (3.2)	34.5 (3.9)	37.7 (6.3)	41.9 NA	32.4 (2.7)	33.7 (3.5)	35.2 NA
	241	67	8	122	21	1	289	55	1

AG/CT	33.6 (2.7)	37.9 (3.4)	41.0 (3.7)	36.3 (2.1)	33.7 NA	NA	30.5 (3.7)	32.1 (3.7)	NA
	104	119	47	29	2	0	139	38	0

GA/TC	37.5 (2.2)	38.4 (2.4)	42.1 NA	32.4 (3.5)	36.9 (3.0)	39.1 NA	33.7 (3.7)	35.6 (2.5)	37.1 NA
	82	117	2	73	31	3	153	54	2

GG/CC	29.5 (4.3)	36.1 (4.6)	44.1 (2.9)	29.2 (3.5)	32.1 (3.0)	29.5 NA	32.7 (3.3)	35.5 (2.5)	NA
	71	150	28	42	16	1	75	13	0

AC/GT	30.8 NA	39.1 NA	NA	32.1 (2.6)	38.0 NA	NA	31.8 (2.7)	35.2 (4.2)	NA
	1	4	0	59	1	0	271	20	0

AT/AT	34.6 (2.9)	37.6 (2.9)	NA	32.4 (3.4)	36.4 NA	NA	33.1 (3.6)	35.8 (3.2)	NA
	109	5	0	110	4	0	139	6	0

GC/GC	34.2 (3.7)	38.7 (2.7)	41.6 (2.3)	26.9 (3.1)	36.5 (2.3)	NA	26.6 (2.2)	30.3 NA	NA
	32	102	12	29	28	0	26	1	0

CA/TG	33.9 (2.5)	38.8 (4.9)	45.8 (3.2)	34.1 (3.0)	35.8 (4.6)	45.0 (3.5)	31.1 (3.9)	35.1 (5.3)	47.7 (3.7)
	54	65	120	72	82	54	158	99	51

CG/CG	37.4 NA	38.2 (2.0)	38.6 (4.1)	NA	NA	NA	38.6 NA	38.6 NA	NA
	3	35	10	0	0	0	1	1	0

TA/TA	35.7 (2.5)	40.5 NA	51.4 NA	38.1 (3.1)	41.1 (2.2)	44.8 NA	31.2 (3.6)	31.3 (3.0)	33.7 NA
	33	2	1	15	16	1	82	29	1

Overall	34.2 (3.8)	37.9 (3.8)	43.9 (4.0)	32.9 (4.0)	36.4 (4.5)	44.4 (4.1)	32.0 (3.5)	34.3 (4.3)	46.9 (4.8)
	730	666	228	551	201	60	1333	316	55

The specifications are as described in the legend for table 3.

**Table 5:** Mean and standard deviation values (given in parentheses, reported only for datasets with size ≥ 5) for slide, for the ten dinucleotide steps, incorporating Watson-Crick basepairs

	Slide values for dinucleotide steps with								

	Minor groove facing the histone octamer			Backbone facing the histone octamer			Major groove facing the histone octamer		
Step	(Region I)			(Region II)			(Region III)		
	BI/BI	BI/BII	BII/BII	BI/BI	BI/BII	BII/BII	BI/BI	BI/BII	BII/BII
AA/TT	-0.2 (0.4)	0.3 (0.6)	1.2 (0.6)	-0.2 (0.4)	0.0 (0.5)	1.3 NA	-0.0 (0.2)	0.1 (0.3)	0.2 NA
	241	67	8	122	21	1	289	55	1

AG/CT	0.2 (0.5)	0.9 (0.4)	1.3 (0.4)	-0.4 (0.3)	0.5 NA	NA	0.2 (0.0)	0.3 (0.6)	NA
	104	119	47	29	2	0	139	38	0

GA/TC	-0.5 (0.3)	0.6 (0.7)	0.5 NA	-0.1 (0.8)	0.7 (0.7)	1.2 NA	-0.2 (0.4)	0.0 (0.3)	0.4 NA
	82	117	2	73	31	3	153	54	2

GG/CC	0.3 (0.4)	0.8 (0.5)	1.4 (0.5)	0.1 (0.5)	0.5 (0.4)	0.5 NA	-0.3 (0.4)	-0.2 (0.6)	NA
	71	150	28	42	16	1	75	13	0

AC/GT	0.1 NA	1.0 NA	NA	-0.5 (0.3)	0.1 NA	NA	-0.5 (0.4)	0.1 (0.3)	NA
	1	4	0	59	1	0	271	20	0

AT/AT	-0.7 (0.3)	-0.2 (0.8)	NA	-0.6 (0.2)	-0.4 NA	NA	-0.6 (0.3)	-0.5 (0.3)	NA
	109	5	0	110	4	0	139	6	0

GC/GC	0.3 (0.5)	0.9 (0.3)	1.0 (0.2)	0.7 (0.3)	0.7 (0.3)	NA	0.7 (0.3)	0.9 NA	NA
	32	102	12	29	28	0	26	1	0

CA/TG	0.3 (0.3)	1.1 (0.8)	2.1 (0.4)	0.5 (0.4)	0.9 (0.5)	2.3 (0.4)	-0.0 (0.6)	0.5 (0.5)	1.8 (0.4)
	54	65	120	72	82	54	158	99	51

CG/CG	1.1 NA	1.4 (0.3)	1.5 (0.2)	NA	NA	NA	0.2 NA	0.5 NA	NA
	3	35	10	0	0	0	1	1	0

TA/TA	-0.4 (0.3)	-0.2 NA	2.1 NA	-0.4 (0.4)	0.2 (0.3)	0.2 NA	-0.3 (0.5)	0.3 (0.5)	0.3 NA
	33	2	1	15	16	1	82	29	1

Overall	-0.1 (0.5)	0.8 (0.6)	1.7 (0.6)	-0.2 (0.6)	0.6 (0.6)	2.1 (0.5)	-0.2 (0.5)	0.2 (0.5)	1.7 (0.6)
	730	666	228	551	201	60	1333	316	55

The specifications are as described in the legend for table 3.

Henceforth, we will refer to the regions where the minor groove of the DNA faces the histone octamer as region I, the regions where the DNA backbone faces the histone octamer as region II, and the regions where the DNA major groove faces the histone octamer as region III.

Owing to the strong bias in the dataset in favour of purine-purine steps, they also dominate the population of each subset, namely, regions I, II and III. However, their proportion in region I is significantly larger (63.8%) as compared to their proportion in the entire dataset (53.0%), while their corresponding proportions are smaller in regions II (42.0%) and III (48.1%). The proportion of purine-pyrimidine steps is significantly smaller in region I (16.3%) as compared to their proportion in the entire dataset (23.2%), while their corresponding proportions are larger in regions II (28.4%) and III (27.2%). In case of pyrimidine-purine steps, their proportion in region I (19.9%) is smaller as compared to their proportion in the entire dataset (23.8%), while their proportion in region II is significantly larger (30.0%) and proportion in region III (24.8%) is similar.

In all three regions, BI/BI conformation is present in the largest proportion, and hence most of the steps take up this conformation most often. However, in region I, GA/TC, GG/CC, GC/GC and CG/CG take up the BI/BII conformation more often than the BI/BI conformation. The CA/TG step is the most uniformly distributed with a significant presence in all three subgroups of all three regions. It takes up the largest share of BII/BII conformation in all three regions.

Roll, twist and slide show correlated variation for the three subgroups in all three regions. Steps with the BII/BII state assume more negative roll, larger twist and large positive slide as compared to steps with BI/BI or BI/BII conformation. Remarkably, the GG/CC step takes up an extremely large and negative mean value of roll for the BII/BII conformation in region I, and correspondingly, a large positive mean slide and high mean rise indicative of stretching, unlike its structure in free DNA as well as other protein-bound DNA.

The nucleosome is expected to assume negative roll values corresponding to a narrow and deep minor groove in region I, and positive roll values corresponding to a wide and shallow minor groove in region III. While this trend holds true overall, the presence of the remarkably flexible CA/TG step in all regions, and the occurrence of BI/BI as well as BII/BII conformation in both regions I and III, leads to some individual steps bucking the trend. Thus the CA/TG step with BI/BI conformation in region I takes up large positive mean roll of 6.6°, while the one with BII/BII conformation in region III takes up large negative mean roll of -6.1°, both values being beyond 1*σ *of the mean value for free B-DNA.

The tilt values also show significant variation, though no obvious trend is seen. The variation in shift is very small, while that for rise is negligible. The exception is the GG/CC step, which in several cases, shows significant variation in shift and rise as well as tilt.

#### Kinks in nucleosomal DNA

An important question of biological relevance is the contribution of the intrinsic, sequence-dependent flexibility of the DNA structure in nucleosome formation, vis-a-vis the role of intrinsic or protein-induced kinks. By definition, a kink should be beyond the elastic limit of dinucleotide step flexibility. The deviation of the various dinucleotide steps from their structure in protein-free B-DNA should provide clues about the number of kinked steps, as compared to the number of steps with deformations within the elastic limit. We observe that the dinucleotide steps in nucleosomal DNA undergo large deviations from their corresponding values in free B-DNA primarily in terms of tilt and roll, which also happen to be the parameters responsible for imparting curvature to a DNA fragment. We have assumed that the structure of a dinucleotide step with both tilt and roll deviating by less than 3*σ *from their mean values in free B-DNA, is within the elastic limits. Hence the structure of a dinucleotide step with tilt or roll deviating beyond 3*σ *of their mean values in free B-DNA qualifies as a kink, formed with or without the assistance of proteins.

Table 6 shows the number of datapoints for each dinucleotide step that deviate by more than 3*σ *from the mean values for either tilt or roll in the free B-DNA dataset [25]. The population has been divided on the basis of orientation with respect to the histone octamer, and further subdivided on the basis of backbone conformation. The total number of kinked steps in the dataset is 421. This amounts to an average of 421/29 ~ 14.5 kinks per structure, or a kink every 144/14.5 ~ 10 steps of the nucleosomal DNA. The largest number (26) of kinks are observed in the structure 1U35, while the smallest number (6) of kinks are observed in the structure 1KX5. Sixteen out of the twenty-nine structures in the dataset have > 14 kinks.

**Table 6:** Total number of datapoints for each dinucleotide step for which the values of tilt or roll lie beyond 3σ of the mean values for these parameters for the free B-DNA dataset, used by Marathe et al. [25]

	Number of kinks of dinucleotide steps with								

	Minor groove facing the histone octamer			Backbone facing the histone octamer			Major groove facing the histone octamer		
Step	(Region I)			(Region II)			(Region III)		
	BI/BI	BI/BII	BII/BII	BI/BI	BI/BII	BII/BII	BI/BI	BI/BII	BII/BII
AA/TT	4 (1.7)	10 (14.9)	4 (50.0)	3 (2.5)	3 (14.3)	1 (100.0)	12 (4.2)	3 (5.5)	0 (0.0)

AG/CT	5 (4.8)	6 (5.0)	4 (8.5)	2 (6.9)	2 (100.0)	NA	26 (18.7)	4 (10.5)	NA

GA/TC	2 (2.4)	13 (11.1)	0 (0.0)	7 (9.6)	3 (9.7)	1 (33.3)	11 (7.2)	6 (11.1)	0 (0.0)

GG/CC	1 (1.4)	27 (18.0)	14 (50.0)	11 (26.2)	3 (18.8)	0 (0.0)	20 (26.7)	5 (38.5)	NA

AC/GT	0 (0.0)	0 (0.0)	NA	1 (1.7)	0 (0.0)	NA	7 (2.6)	3 (15.0)	NA

AT/AT	0 (0.0)	0 (0.0)	NA	0 (0.0)	1 (25.0)	NA	4 (2.9)	0 (0.0)	NA

GC/GC	3 (9.4)	6 (5.9)	2 (16.7)	1 (3.4)	1 (3.6)	NA	3 (11.5)	0 (0.0)	NA

CA/TG	1 (1.9)	7 (10.8)	62 (51.7)	7 (9.7)	7 (8.5)	13 (24.1)	41 (25.9)	13 (13.1)	3 (5.9)

CG/CG	0 (0.0)	0 (0.0)	0 (0.0)	NA	NA	NA	0 (0.0)	0 (0.0)	NA

TA/TA	0 (0.0)	0 (0.0)	1 (100.0)	0 (0.0)	0 (0.0)	0 (0.0)	11 (13.4)	10 (34.5)	0 (0.0)

Overall	16 (2.2)	69 (10.4)	87 (38.2)	32 (5.8)	20 (10.0)	15 (25.0)	135 (10.1)	44 (13.9)	3 (5.5)

The cutoff values used are: tilt < -7.2° or tilt > 6.0°, and roll < -14.7° or roll > 17.1°.

The numbers in parentheses denote the population as percentages, of the total number of datapoints for the respective step in the given conformation. The rest of the specifications are as described in the caption for table 3.

Of the 421 steps assuming a kinked structure, 235 have only the roll value deviating by 3*σ *from its free B-DNA mean value, while 153 steps have only the tilt value deviating by 3*σ *from its free B-DNA mean value. Nearly 40% of the steps in region I with backbone conformation BII/BII, and 25.0% of the steps in region II with backbone conformation BII/BII, are kinked, with CA/TG contributing a large share in both cases. In region III, significant proportions of CA/TG and GG/CC steps with BI/BI backbone conformation, and TA/TA and GG/CC steps with BI/BII backbone conformation, are kinked.

In region I, 150 of the 172 kinked steps take up negative roll value as expected. Of the 22 steps with positive roll, 3 AG/CT steps and 1 CA/TG step take up positive roll beyond 3*σ *of its free B-DNA mean value, accompanied by a BI/BI backbone conformation. Of these, the CA/TG kink in the vicinity of SHL -5.5 in the structure 1M1A, and the AG/CT kink in the vicinity of SHL -1.5 in the structure 1U35, are compensated by larger and opposite kinks at the neighbouring steps. However, the AG/CT kink in the vicinity of SHL 6.5 in the structure 1M1A, and the AG/CT kink in the vicinity of SHL 4.5 in the structure 1S32, are not compensated for.

In region III, 162 of the 182 kinked steps take up positive roll. Of the remaining 20 steps, 3 CA/TG steps take up negative roll beyond 3*σ *of its free B-DNA mean value, accompanied by a BII/BII backbone conformation. All the 3 steps occur at the edge of the major groove region centered at SHL -6, in the structures 1P3F, 1P3O and 2F8N. These kinks are compensated by large positive roll angle values at the neighbouring step, as well as at the step adjacent to it.

In region II, 41 of the 67 kinked steps have only the tilt value deviating by 3*σ *from its free B-DNA mean value, while 22 kinked steps have only the roll value deviating by 3*σ *from its free B-DNA mean value. Thus tilt contributes nearly twice the share of kinks in this region as compared to roll.

The intra-basepair parameters propeller twist, buckle and open angle of the basepairs constituting the dinucleotide steps with kinks are far more likely to take up values more than 1*σ *away from the mean nucleosome dataset values for these parameters, as compared to the proportion over the entire nucleosome dataset. Over the entire dataset, only 25-30% of basepairs take up propeller twist, buckle or open angle values beyond 1*σ *of the mean value for these parameters, but within the kinked steps, 60-70% of steps have one or both the constituent basepairs assuming values beyond 1*σ *of the mean value. In case of other basepair parameters such as the two glycosidic angles, and the C_1_'-C_1_*' *and C_8_-C_6 _separations, the proportions are similar to those for propeller twist, buckle and open angle. The basepair parameters seldom assume values beyond the 2*σ *limit.

### Variation within and across structures

#### At dinucleotide level

**Figure 3:**
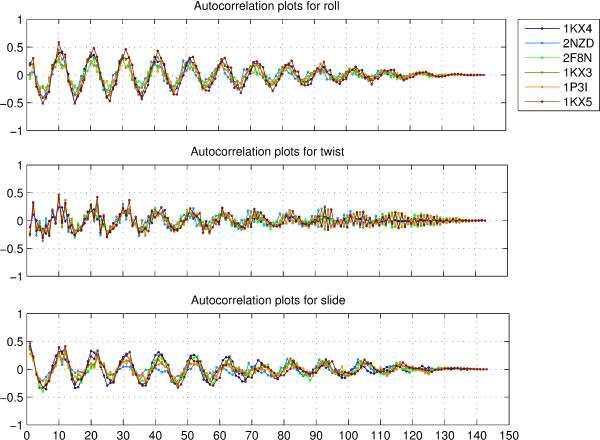
**Autocorrelation plots for roll, twist and slide**. Autocorrelation values for the rotational parameters roll and twist, and the translational parameter slide, for the six structures with PDB id's 1KX4, 2NZD, 2F8N, 1KX3, 1P3I and 1KX5, corresponding to sequences 1, 2, 3, 4, 5 and 6 respectively.

It must be emphasized that while there are similarities within and across structures in the distribution of parameters at the dinucleotide step level, there are also substantial differences. The trajectories for the dinucleotide step parameters, for the six structures, namely, 1KX4, 2NZD, 2F8N, 1KX3, 1P3I and 1KX5, corresponding to sequences 1, 2, 3, 4, 5 and 6 respectively, are shown in figures S1-S6 of additional file 1. We have represented only six structures for clarity, however, the observations remain valid for the entire dataset of twenty-nine structures. The differences within the same structure become evident when one looks at the autocorrelation values for different parameters in a structure. Figure 3 shows the autocorrelation values for the parameters roll, twist and slide, for the six representative structures. The autocorrelation plots for tilt, shift and rise are shown in figure S7 of additional file 1. It is immediately apparent that roll and slide display strong periodicity throughout each structure with a period just greater than 10. However, the peaks for strong correlation or anticorrelation are not always at the same position with respect to SHL 0, for different structures, and in many cases, are distributed over two or three positions. Twist shows a weak periodicity, and the other three parameters display no periodicity. This validates the theoretical prediction by Bishop [19] that tilt, shift and rise are allowed relatively greater freedom in the nucleosome structure.

**Figure 4:**
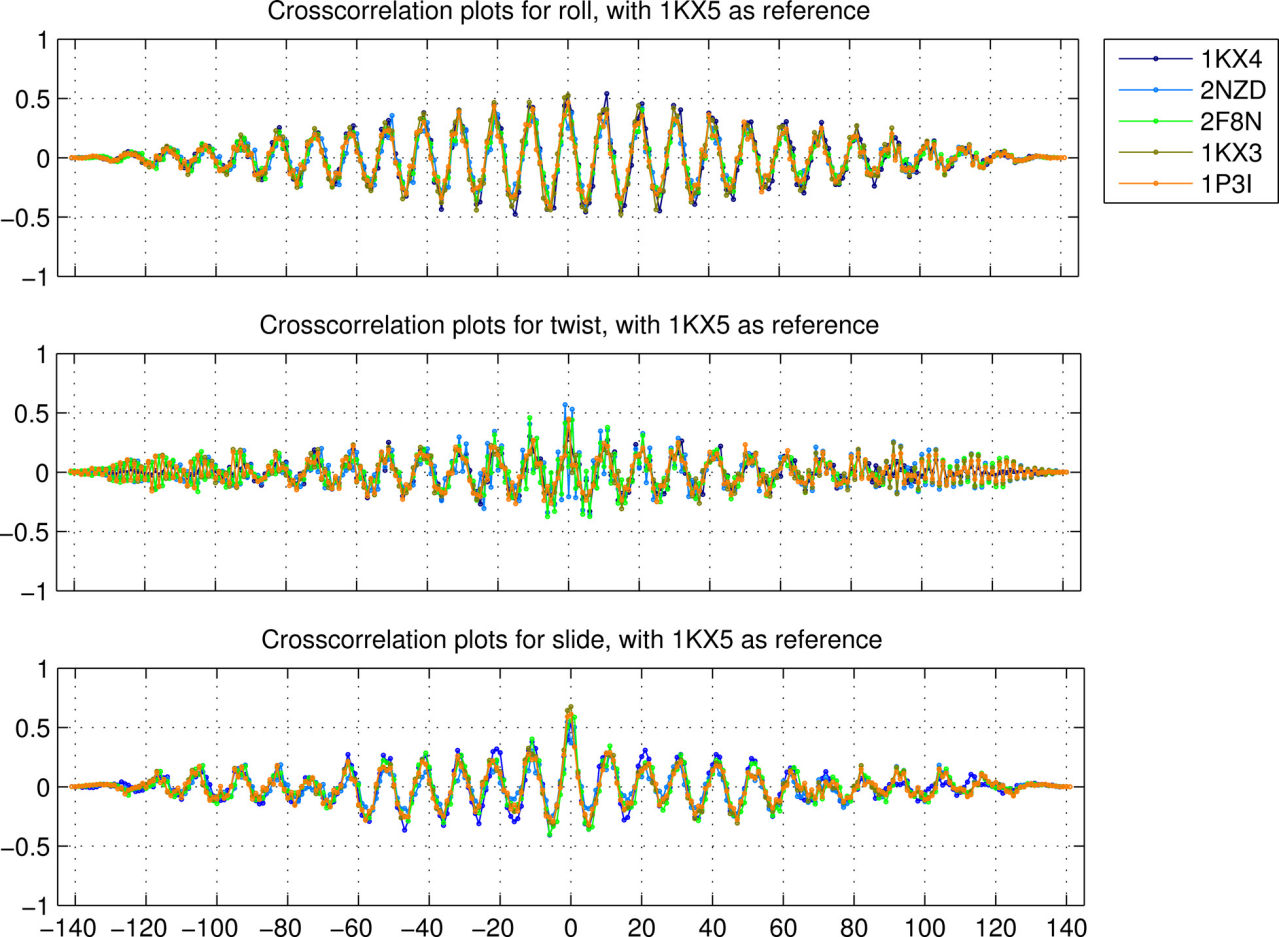
**Crosscorrelation plots for roll, twist and slide**. Crosscorrelation values for the rotational parameters roll and twist, and the translational parameter slide, for the five structures with PDB ids 1KX4, 2NZD, 2F8N, 1KX3 and 1P3I, corresponding to sequences 1, 2, 3, 4 and 5, with respect to the corresponding parameters for the best resolved crystal structrure of the nucleosome with PDB id 1KX5, corresponding to sequence 6.

The differences across structures become evident when one looks at the crosscorrelation plots for different dinucleotide step parameters. Figure 4 shows the crosscorrelation values for the parameters roll, twist and slide, for the five structures 1KX4, 2NZD, 2F8N, 1KX3 and 1P3I, corresponding to sequences 1, 2, 3, 4 and 5, with respect to the parameters for the best resolved crystal structrure 1KX5, corresponding to sequence 6. The corresponding plots for tilt, shift and rise are shown in figure S8 of additional file 1. The crosscorrelation peaks for each structure seem to be displaced by few positions as compared to the peaks for any other structure. In case of both autocorrelation and crosscorrelation plots, the distribution for slide seems more periodic than that for roll.

#### At trinucleotide level

**Figure 5:**
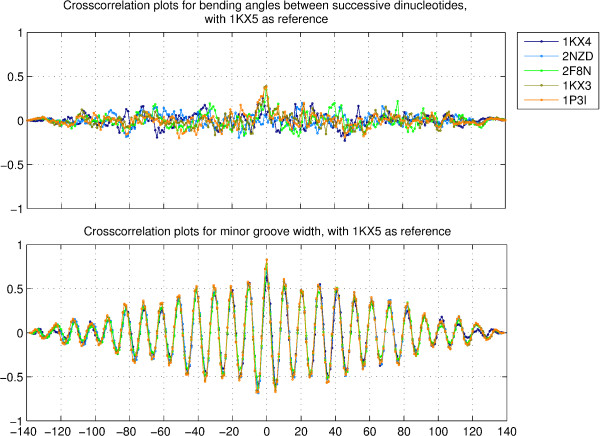
**Crosscorrelation plots for successive bending angles and minor groove width**. Crosscorrelation values for the successive bending angles, and the minor groove width, for the five structures with PDB id's 1KX4, 2NZD, 2F8N, 1KX3 and 1P3I, corresponding to sequences 1, 2, 3, 4 and 5, with respect to the corresponding parameters for the best resolved crystal structrure of the nucleosome with PDB id 1KX5, corresponding to sequence 6.

Successive bending angles are proportional to the difference of roll angles of consecutive, overlapping dinucleotide steps (data not shown) and as such, are indicators of trinucleotide level structural variation. Figure S9 in additional file 1 shows the trajectories for the successive bending angles for the six representative structures, while Figure 5 (upper panel) shows the crosscorrelation values for the successive bending angles, for the five structures 1KX4, 2NZD, 2F8N, 1KX3 and 1P3I, with respect to the corresponding parameters for the structrure 1KX5. It is clearly seen that even at zero lag, the plots show a very small peak, and there is no correlation at non-zero lag. This clearly indicates that the pattern of local bending is very distinct across different structures, despite very similar sequences for all the structures, except 1KX4. The plots for autocorrelation values of successive bending angles in the six structures mentioned above (upper panel of figure S11, additional file 1) also display an irregular pattern, implying that even different regions within the same structure show different bending profiles.

The minor groove width calculation spans three basepairs and in that sense, is a trinucleotide parameter. Figure S10 in additional file 1 shows the minor groove width trajectories for the six representative structures, while Figure 5 (lower panel) shows the crosscorrelation plots for minor groove width values. In contrast to successive bending angles, the variation in minor groove width is seen to be uniform, strongly periodic, and shows very similar pattern across all structures.

#### At octanucleotide and decanucleotide levels

**Figure 6:**
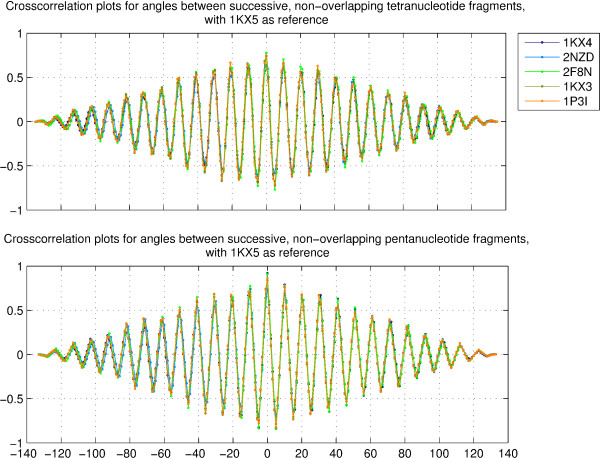
**Crosscorrelation plots for angles between tetranucleotide and pentanucleotide fragments**. Crosscorrelation values for the angles between global helix axes fitted to backbone C_1_*' *atoms of successive, non-overlapping tetranucleotide fragments, and the angles between global helix axes fitted to backbone C_1_*' *atoms of successive, non-overlapping pentanucleotide fragments, for the five structures with PDB id's 1KX4, 2NZD, 2F8N, 1KX3 and 1P3I, corresponding to sequences 1, 2, 3, 4 and 5, with respect to the corresponding parameters for the best resolved crystal structrure of the nucleosome with PDB id 1KX5, corresponding to sequence 6. For the purpose of calculation, the value of the angle was assigned to the 4*^th^*/5*^th ^*position in the octanucleotide/decanucleotide. The angles are assigned the sign of their dot product with the average of vectors in the x-directions of the two central basepairs.

Figure 6 (upper panel) shows the crosscorrelation plots for the angles between the global helix axes fitted to successive, non-overlapping tetranucleotide fragments. It is clearly seen that at the octanucleotide level, the bending between successive, non-overlapping tetranucleotide fragments shows a periodic variation across all structures, independent of differences in sequence. This uniformity of variation of the bending angle across structures with different sequences becomes even more pronounced when one considers crosscorrelation values for the angles between global helix axes fitted to successive, non-overlapping pentanucleotides (as seen by the sharper peaks of larger magnitude in the lower panel of Figure 6), since this region corresponds to approximately one turn of the helix. This observation validates the theoretical prediction by Bishop [19] regarding this length scale. Thus within one turn, the longer length scales from octanucleotide to decanucleotide display the same trend of bending across all structures. It is possible that this trend carries over to even longer length scales.

The trajectories for the angles between tetranucleotide fragments, and the angles between pentanucleotide fragments, for the six representative structures, as well as the autocorrelation plots for the angles are shown in figures S12, S13 and S14 respectively, in additional file 1. The periodicity for these two parameters within each structure is clearly evident.

### Curvature at a length scale of half a superhelical circle

DNA curvature at longer length scales gives an idea as to whether the similarity of bending at a length scale of one turn (~ten nucleotides) adds up at longer length scales. Table 7 shows the mean and standard deviation values of d/l*_local_*, I*_max_*/I*_min_*, (I*_max _*+I*_mid_*)/I*_min _*and radius of curvature for successive, overlapping 36-mer fragments of each structure. A 36-mer fragment is just short of half the superhelical circle. It is clearly seen that the mean values for d/l*_local_*, I*_max_*/I*_min_*, (I*_max _*+I*_mid_*)/I*_min _*and radius of curvature for all structures are within 1*σ *deviation of each other indicating that most of the fragments have similar curvature. However, the standard deviation values being ~4-5% of the mean ROC indicates that the variation in curvature of individual fragments of the same structure or between different structures is not negligible.

**Table 7:** The mean and standard deviation values (in parentheses) for each parameter quantifying curvature, calculated for all successive, overlapping 36-mer fragments (excluding the terminal basepairs) in each individual structure.

PDB id	d/l*_local_*	I*_max _*/I*_min_*	$\frac{{\text{I}}_{max}+{\text{I}}_{mid}}{{\text{I}}_{min}}$	ROC (Å)
1KX4	0.707 (0.018)	5.9 (0.5)	11.1 (1.1)	41.9 (2.0)
2NZD	0.707 (0.020)	5.8 (0.5)	11.0 (1.0)	41.8 (2.0)
1U35	0.710 (0.017)	5.9 (0.5)	11.1 (1.1)	42.0 (1.9)
2F8N	0.714 (0.017)	5.9 (0.5)	11.1 (1.1)	41.9 (2.0)
1AOI	0.710 (0.019)	5.9 (0.5)	11.0 (1.0)	42.0 (2.0)
1KX3	0.707 (0.020)	5.9 (0.5)	11.0 (1.0)	41.7 (2.0)
2CV5	0.708 (0.017)	5.9 (0.5)	11.1 (1.0)	41.9 (1.9)
1EQZ	0.712 (0.018)	5.9 (0.5)	11.1 (1.0)	41.8 (2.0)
1F66	0.708 (0.016)	5.8 (0.5)	11.0 (1.0)	41.9 (2.1)
1M18	0.713 (0.017)	5.9 (0.5)	11.1 (1.0)	42.2 (2.0)
1M19	0.716 (0.017)	6.0 (0.5)	11.2 (1.1)	42.0 (1.9)
1M1A	0.712 (0.017)	5.9 (0.5)	11.1 (1.0)	42.0 (1.8)
1P34	0.708 (0.019)	5.9 (0.5)	11.1 (1.1)	42.0 (2.1)
1P3A	0.708 (0.019)	5.9 (0.5)	11.0 (1.0)	41.9 (2.0)
1P3B	0.710 (0.019)	5.9 (0.5)	11.1 (1.0)	41.8 (2.0)
1P3F	0.710 (0.019)	5.9 (0.5)	11.1 (1.0)	41.8 (2.0)
1P3G	0.709 (0.019)	5.9 (0.5)	11.1 (1.0)	42.0 (2.0)
1P3I	0.708 (0.019)	5.9 (0.5)	11.1 (1.0)	41.9 (2.0)
1P3K	0.705 (0.020)	5.9 (0.5)	11.0 (1.0)	41.9 (2.0)
1P3L	0.710 (0.019)	5.9 (0.5)	11.1 (1.0)	42.0 (2.0)
1P3M	0.711 (0.020)	5.9 (0.5)	11.1 (1.0)	42.0 (2.0)
1P3O	0.708 (0.020)	5.9 (0.5)	11.0 (1.0)	41.9 (2.0)
1P3P	0.709 (0.019)	5.9 (0.5)	11.1 (1.0)	41.9 (2.0)
1S32	0.709 (0.019)	5.9 (0.4)	11.0 (0.9)	41.9 (1.9)
1ZLA	0.708 (0.019)	5.9 (0.5)	11.0 (1.0)	41.8 (2.0)
2NQB	0.708 (0.020)	5.8 (0.5)	11.0 (1.1)	41.9 (2.1)
3C1B	0.706 (0.019)	5.9 (0.5)	11.1 (1.0)	42.0 (2.0)
1KX5	0.714 (0.018)	6.0 (0.6)	11.2 (1.1)	41.9 (2.0)
2PYO	0.712 (0.019)	5.9 (0.6)	11.2 (1.1)	41.9 (2.1)

## Discussion

### Nucleosomal DNA is B-like at the dinucleotide step level

An analysis of the twenty-nine nucleosome X-ray crystal structures of better than 3 Å resolution reveals significant dinucleotide level structural variability in nucleosomal DNA, despite limited variation in sequence, with only six unique sequences, of which only one differs significantly from the other five. A survey of the dinucleotide step parameters indicates that all the dinucleotide steps in the dataset assume values characteristic of B-form DNA. Specifically, the parameter Z*_p_*, which has been shown to be the most reliable indicator of A-versus-B discrimination at the dinucleotide step level [25,28], indicates that with the exception of ten steps, the entire nucleosomal dataset is B-like. This observation is in contrast to the study of all nucleosomal DNA structures reported by Xu and Olson [29]. Xu and Olson have classified nucleosomal dinucleotide steps with large positive roll (> 7°) and negative slide (< -1 Å) (as calculated by the 3DNA program [30]) as exhibiting an "A-type kink-and-slide geometry", and observe 15% kink-and-slide steps assuming this geometry. However, it has been shown that roll is not a reliable indicator of an A-like geometry [25]. Further, of the 421 nucleosomal DNA steps in our dataset with at least one of tilt or roll assuming values beyond 3*σ *of the free B-DNA mean values, only 16 steps (proportion 3.8%) assume roll value > 7° and slide value < -1 Å.

The presence of the non-canonical BII conformation in nucleosomal DNA in proportions larger than those observed in other protein-bound DNA highlights its role in modulating the nucleosomal DNA structure by facilitating a bend into the minor groove. In the free B-DNA dataset, only the CA/TG step has significant proportion of datapoints with one or both strands in BII conformation, which might explain why the CA/TG step is often strategically placed at positions where the DNA bends into the minor groove [18,20]. This also explains why the CA/TG step constitutes the largest share of steps with at least one strand in BII conformation, in all regions of the nucleosome.

A comparison of the mean values for the three parameters, namely, roll, twist and slide, which contribute predominantly to the DNA curvature and superhelical pitch, for the nucleosome dataset with those for the free B-DNA dataset [25] sheds some light on the role played by the histone proteins. The mean values for BI/BI conformation are within 1*σ *of the mean values for free B-DNA, while most of the mean values for BII/BII and few of the mean values for BI/BII conformation are beyond 1*σ *of the corresponding mean values for free B-DNA. As a result, the overall mean values for roll, twist and slide for the BII/BII conformation in all three regions are beyond 1*σ *of the free B-DNA mean values, and reinforce the importance of the BII/BII conformation in modulating the nucleosome structure.

### The kinks into the minor groove at GG/CC steps may be influenced by proteins and environmental factors

A minor groove kink is generally characterised by large negative roll, large twist and large positive slide, along with a state 7 backbone conformation i.e. a BII/BII conformation for *ϵ*-ζ and canonical B-DNA values for the other backbone torsion angles. The negative mean roll angle value with extremely large magnitude for the GG/CC steps occurring in region I and taking up a BII/BII backbone conformation, and the significant proportion of kinks among these steps prompted us to individually examine all the steps. We observe at least one kinked GG/CC step in all structures with the exception of 1KX5, 1M18, 1M19, 1M1A, 2CV5 and 2PYO. However, it must be pointed out that not all these extreme values belong to a step with state 7 conformation, in many cases, the step assumes one of the other six conformations in one or both of the strands. In most of the cases, these GG/CC steps occur at SHL -1.5 and/or +1.5 with respect to the pseudo two-fold axis of the superhelix. While many of these kinks into the minor groove are also accompanied by a stretching of the DNA as described earlier [31], in some cases the kinked conformation is observed in the absence of any stretching. Several GG/CC steps with a sharp kink also have an accompanying large positive slide. Thus at SHL -1.5 in the structure 1F66, the roll value is -68.1° with corresponding tilt, twist, shift, slide and rise values of -4.3°, 43.0°, 0.3 Å, 1.2 Å and 4.7 Å respectively, while the GG/CC steps in 1KX4 at SHL ±1.5 provide examples of distortion without stretching, but with positive slide values.

In about half the reported structures, a GC/GC step adjacent to the GG/CC step is also kinked. For example, while the GG/CC step at location -1.5 in the structure 1KX3 assumes tilt, roll, twist, shift, slide and rise values of 7.5°, -26.1°, 43.3°, -0.4 Å, 0.4 Å and 4.0 Å respectively, the neighbouring GC/GC step assumes values of -15.5°, -17.0°, 37.3°, 0.6 Å, 0.5 Å and 4.6 Å respectively. In the other structures, the neighbouring GC/GC step tends to compensate for the unusual conformation of the GG/CC step. On the other hand, the GG/CC step at SHL -1.5 has no significant distortion in some structures, while the neighbouring GC/GC step is extremely distorted. This is further proof of the variability observed even across structures with identical sequences. It must be noted that the nucleotides in these GG/CC steps with extreme values of roll, twist and slide were not hydrogen bonded to any amino acids.

Similarly, several of the AA/TT steps also assume large negative roll values at locations where the minor groove faces the histone octamer. This is observed in eleven of the structures at one or two locations, the most prominent being SHL ±4.5, and in few cases at SHL ±3.5 and ±1.5. Many of these AA/TT steps are observed to be part of A-tracts. Some of these are again accompanied by large twist and large postive slide values. For example, in the structure 2F8N, the roll, twist and slide values of the AA/TT step at SHL -3.5 are -14.2°, 44.6° and 1.7 Å respectively, while those for the step at SHL 1.5 are -19.5°, 44.0° and 1.8 Å respectively. While these steps also have an abnormal tilt, most of them do not display a large rise characteristic of stretching.

In addition to the above steps, few of the AG/CT steps are also observed to assume large negative roll, large twist and large positive slide at SHL 2.5 and in some cases, at SHL -1.5. For example, in the structures 1M1A and 2CV5, the tilt, roll, twist, shift, slide and rise values are 7.4°, -16.6°, 45.0°, 0.6 Å, 1.8 Å and 3.6 Å, and 4.7°, -18.5°, 40.6°, 1.1 Å, 1.6 Å and 3.5 Å respectively at SHL -1.5.

A comparison of the minor groove kinks in the nucleosome dataset with those observed in the DNA bound to other proteins shows that similar kinks are also associated with CA/TG steps in the Cre recombinase-bound DNA and the I-Cre I homing endonuclease-bound DNA structures. A dinucleotide step with large positive slide in the non-nucleosomal protein-bound DNA is most likely to be CA/TG, as noted by Tolstorukov et al. [18]. The only non-CA/TG dinucleotide steps with a slide value > 2.0 Å are an AA/TT step in the hyper-thermophile SAC 7D-DNA complex structure with PDB id 1WTQ [32] and a CG/CG step in the catabolite activator protein-bound DNA structure with PDB id 1O3R [33,34]. While both these steps assume large positive slide, they do not assume large negative roll and hence are not equivalent to a kink into the minor groove. The absence of a minor groove kink at the TA/TA steps in the crystal structure datasets of either the nucleosomal DNA or the non-nucleosomal protein-bound DNA is surprising, since it has been suggested earlier [35] that a minor groove kink at the TA/TA step will be energetically less costly as compared to an equivalent kink at the CA/TG step.

However, while the contribution by the CA/TG step to curvature and superhelical pitch remains largest, it is not exclusively confined to it. Given that the GG/CC step favours positive roll, small twist and negative slide [25] in free DNA, the frequency with which it is observed kinking into the minor groove in the nucleosome dataset is intriguing. The observation that most of the distorted GG/CC steps are observed at SHL ±1.5 indicates that this position might be of special relevance [31] and any dinucleotide step around this region might be vulnerable to stretching and distortion. However, exactly which step gets kinked and/or stretched might depend on a combination of factors such as the position, the dinucleotide sequence and the differing context of the nucleosome within chromatin. The kinks into the minor groove at GG/CC steps might also point to a more general tendency to have a mixture of favourable and unfavourable sequences, which results in only marginally stable nucleosomes [14], so that the nucleosome can be disrupted during transcription and replication while simultaneously preventing inappropriate access.

### Extreme kinks into the major groove are less likely as compared to extreme kinks into the minor groove

The kinks into the major groove do not have tilt or roll values deviating too far from 3*σ *of the free B-DNA mean values. Only 4 out of 182 kinks in region III take up positive roll with a value deviating by > 5*σ *from the mean free B-DNA value, while the corresponding number for kinks in region I is 27 out of 172. The four steps are: an AT/AT step at SHL -1 in the structure 1EQZ (similar to the GG/CC kink into the minor groove, this is an unlikely conformation, since the AT/AT step favours nearly zero roll and slide in free B-DNA and other protein-bound DNA [25]), a GG/CC step at SHL -6 in the structure 1P3B, a CA/TG step at SHL -2 in the structure 2F8N and a CA/TG step at SHL -1 in the structure 2NZD.

A comparison of the major groove kinks in the nucleosome dataset with those observed in the DNA bound to other proteins shows that similar kinks are also observed at a variety of steps in different structures such as hyperthermophile SAC 7D-bound DNA (CG/CG, TA/TA, AA/TT), LAC-repressor-bound DNA (CG/CG), catabolite activator protein-bound DNA (CA/TG, CG/CG, GA/TC), integration host factor-bound DNA (AA/TT), Eco RV endonuclease-bound DNA (TA/TA), *γδ *resolvase-bound DNA (TA/TA) and TATA binding protein-bound DNA (TA/TA, AA/TT and AG/CT). Unlike most of the kinks in the nucleosome structures, these kinks are extremely sharp (roll ~50-60°) and often accompanied by a large rise of > 4.0 Å and sometimes by a small twist. However, similar to the conformation observed in nucleosomes, most of these steps are also B-DNA like in terms of Z*_p_*, slide and the backbone conformational parameters.

### The linear elastic model may not be applicable to nucleosomal DNA

A number of algorithms, based on the simple harmonic approximation, have been developed to predict the energetic cost of nucleosome formation [18,20-24]. These algorithms typically assign the parameter mean values in the X-ray crystal structure dataset of protein-DNA complexes (or values obtained by minimising the conformational energy [24]) as 'zero energy' values, and use a quadratic term as energy penalty for deviations from these mean values. However, analysis of high-resolution free and protein-bound DNA crystal structures [25] indicates that even within the B-DNA family, steps such as CA/TG assume a trimodal distribution in case of several dinucleotide parameters. Multimodal distribution of twist and slide values for several steps has been observed in molecular dynamics simulations carried out by the Ascona B-DNA consortium [26]. In case of such steps, use of distribution mean values is invalid. In this context, it must also be pointed out that our definition of a kink in terms of mean and standard deviation values for tilt and roll over the entire free B-DNA dataset is meaningful only because the values for both parameters assume a single Gaussian distribution over the entire dataset.

The presence of a kink, on average, over every turn of the nucleosomal DNA helix poses an additional problem for algorithms based on the linear elastic model. Molecular dynamics studies [36] have shown that kinks similar to the ones observed in the nucleosome are stiff. Hence the simple harmonic approximation may lead to an incorrect value for energy of formation of a kink, and consequently, for energy of nucleosome formation. This is in agreement with the observation by Sereda and Bishop [37] that "removal of the largest amplitude deformations in the nucleosome had a significant [positive] effect on all elastic rod models" and therefore "a simple linear approximation does not properly capture the material properties of DNA".

### Distribution of trinucleotide parameters indicates a more uniform variation in slide as compared to that in roll

The correlation values for successive bending angles within and across structures indicate that the bending profile fluctuates significantly not only for structures with different sequences but also for those with identical sequences, as well as for different regions within a structure. This variation in successive bending angle values can be most clearly explained in terms of the pattern of roll angle values. In all the structures, we observe blocks of two or three steps with negative roll values in the regions around SHL ±i.5 (i = 1, 2, 3, 4, 5, 6), followed by a junction step with nearly zero roll and a block of two or three steps with positive roll in the regions around SHL ±i (i = 1, 2, 3, 4, 5, 6, 7). This has been discussed by Richmond and Davey [17] for the structure 1KX5. However, within the block of negative roll, any of the two or three steps can have the highest magnitude of roll, and this step is observed to be different even for different structures corresponding to the same sequence. The observation also holds true for blocks of positive roll. This explains the difference in pattern of successive bending angles across structures corresponding to different as well as identical sequences, and highlights the structural versatility of B-form DNA.

It was shown by Richmond and Davey [17] in the structure 1KX5 that the regions between SHL -3 and SHL +3 display smooth bending when the minor groove faces the histone octamer while the minor groove blocks facing the histone octamer and farther away from the dyad are kinked with large negative roll angle values. However, this observation does not hold true in general, as seen by a survey of the successive bending angle values in all structures. While the structures in the 1M1 series display smooth bending in the region between SHL -3 and +3 and kinks outside those blocks, most of the other structures have sharp kinks throughout in blocks of both positive and negative roll. Even in 1M1A, there are sharp kinks at SHL -1.5 and -2. Several structures have a kink at -1.5 or +1.5 (1EQZ) as described earlier while some of them have kinks at -2 or +2. It must be noted that the structure 1KX5 also has a sharp kink at +2. As a result of variation in kinks versus smooth bending, the variation in the shift parameter is also not uniformly different for the regions binding H3 and H4 as against the regions binding H2A and H2B, as noted for 1KX5 [17].

A survey of the successive bending angle values for all structures also indicates that for almost all of the 146-basepair structures, the kinks in the shorter half (please refer 'Methods' section for definitions of the shorter and longer halves) of the structures are larger in magnitude than the corresponding kinks in the longer half. This trend is seen most consistently in structures of the 1P3 series but it is also observed in all other 146-basepair structures. In addition, most of the large kinks into the minor and major grooves, commented upon in the previous section, occur in the shorter half of the DNA structure. These two observations together imply that stretching in one half of the structure to cover the same distance with one basepair less as compared to the longer half leads to sharper kinks throughout the shorter half. Of the three 145-basepair structures, only 2F8N is observed to assume sharper kinks in the first half compared to the second half of the structure. The 147-basepair structures do not display this behaviour.

In contrast to successive bending angles, the variation in minor groove width is seen to be far more consistent across all structures. Minor groove width has been shown to be proportional to the mean of the slide of the two dinucleotide steps constituting the trinucleotide [38]. The consistency of the minor groove width variation across different nucleosome structures implies that unlike roll, the slide parameter adds up in a regular fashion at the trinucleotide level, independent of sequence. This observation supports the earlier suggestion that slide is the most important parameter in determining DNA superhelical structure [18].

### Interplay of slide, roll and twist causes variation of gross structural parameters at different length scales

We observe that at a length scale of thirty-six basepairs, curvature values have large fluctuations, as indicated by the standard deviation values being ~4-5% of the mean ROC. This is the cumulative effect of large variation in roll angles at the same position with respect to SHL 0. While roll is primarily responsible for curvature, twist and slide are responsible for superhelical rise. However, within and across structures, slide displays more regular variation as compared to roll and twist. Thus the pattern of interplay between these parameters is different at varying length scales, for long fragments within the same structure and also across structures, leading to variations in the core nucleosome structure. This observation is in agreement with results from Bishop's analysis of crystal structures and molecular dynamics simulation data of the nucleosomal DNA [19].

### Structural versatility of B-form nucleosomal DNA may contribute to the plasticity of gene expression

Nucleosome positioning is known to be controlled by various factors such as preference of the DNA sequence to assume a nucleosome like structure, DNA methylation, higher order chromatin structure and presence of DNA binding proteins such as transcription factors [3]. Of these factors, the intrinsic preferences of the DNA sequence have been shown to play a key role in determining the organisation of nucleosomes *in vivo *[5-7]. There have been a host of studies which have attempted to derive the complete sequence pattern characteristic of nucleosomal DNA by analysing the in-phase and out-of-phase occurrences of various dinucleotides such as AA and TT, GG and CC, AT, TA, and CA and TG [4,6,8-11]. In this study, we have not looked at sequence variation in the nucleosome crystal structure dataset, as it has only two widely differing sequences. However, it must be noted that the statistical enrichment of preferred dinucleotide and longer motifs is observed to occur only modestly above a random distribution and is limited to nucleosomes immediately upstream and down-stream of a transcription start site (TSS) [12-14]. In other words, formation of a majority of *in vivo *nucleosomes is largely controlled by factors other than the DNA sequence. This is especially true for nucleosomes in the vicinity of genes, which display a higher plasticity in terms of variation in their expression, with such nucleosomes displaying a more homogeneous and dynamic occupancy across promoters, and a particularly high occupancy close to the TSS [39]. We would like to propose that the large range of permissible variation in structure of B-form DNA [25] acts as an important factor in the formation of nucleosomes in such regions. There has not been any focus on this factor, because we do not have the structure of nucleosomal DNA in *in vivo *conditions, and it is difficult to comment on its variability. However, our analysis of all the available nucleosome crystal structures shows that even within this limited dataset, and even for the same sequence, there is an ensemble of dinucleotide and trinucleotide level B-form structures, that can lead to similar core nucleosome structure. We also hypothesise that the structural versatility of nucleosomal DNA might act as an important facilitator of expression plasticity by changing the volume of periodically exposed grooves and thereby, varying the probability of recognition by regulatory proteins that bind to these grooves [14,40,41].

### The best resolved nucleosome crystal structure may not be the 'ideal' template

Several studies have focussed on developing algorithms to predict the energetic cost of nucleosome formation [18,20-22] by using as a template, the best resolved X-ray crystal structure of nucleosome with PDB id 1KX5 [15]. We see major drawbacks in this approach, since our analysis clearly points at significant variation in local nucleosomal structure, and hence it seems unlikely that the single static structure represented in 1KX5 is *the *structure for nucleosomal DNA. This is in agreement with the observation by Xu and Olson [29] that "Nucleosomal DNA can also take slightly different conformational routes in the course of its packaging" and "the different nucleosomal pathways accommodate the deformations of a common sequence ... in different ways". Given that DNA curvature is essentially statistical [42-44], and considering that statistical and static averages are often different [45,46], the structure 1KX5 is unlikely to represent the statistical mean of such an ensemble. Hence calculation of the energetic cost for a given genomic sequence to take up a nucleosome structure, assuming the structure of 1KX5 as the template, may not lead to biologically meaningful results.

## Conclusions

The nucleosomal DNA structure, despite very limited sequence variation in available experimental data, displays significant variation at the dinucleotide step level for parameters such as roll, twist and slide, but remains within the B-DNA family. We also observe a large number of kinks in the nucleosomal DNA structure. Extreme kinking into the minor groove is more frequent than extreme kinking into the major groove. Particularly at SHL ±1.5, the GG/CC step, which favours a conformation with positive roll, small twist and negative slide in free B-DNA [25], is frequently observed to assume large negative roll, leading to a kink into the minor groove. This indicates a possible indirect role for proteins and the environment. The multimodal distribution of dinucleotide parameters for some steps [25,26] and the presence of a large number of kinks in the nucleosomal DNA structure may lead to an incorrect value of nucleosome formation energy, as predicted by algorithms which use the linear elastic model [18,20-24] for this calculation.

At the trinucleotide level, while roll does not add up to give a consistent pattern for local bending angle, slide adds up consistently in a position specific fashion even across widely differing sequences to give similar variation in minor groove width. This highlights the role of slide as being the principal dinucleotide parameter in characterising nucleosomal DNA structure. At longer length scales of octanucleotide and decanucleotide, the pattern of bending is consistent across all sequences. At a length scale of thirty-six basepairs, the curvature is generally uniform with small variation, within and across structures.

Overall, our results indicate that there is an ensemble of dinucleotide and trinucleotide level parameters that can give rise to similar core nucleosome structure. This structural versatility may act as a significant factor influencing the formation of nucleosomes in the vicinity of high-plasticity genes, and in varying the probability of binding by regulatory proteins. In view of this structural versatility, use of the best resolved X-ray crystal structure of the nucleosome [15] as *the *template for predicting the energetic cost of nucleosome formation by potential nucleosome sequences [18,20-22], appears questionable.

**Note:** 2.5 Å resolution X-ray crystal structures of two distinct 145-basepair sequences, belonging to the so-called '601' fragment, were reported by Vasudevan et al [47] while this manuscript was under review. Hence we now have crystal structures of the nucleosome corresponding to four widely differing sequences. A quick survey of the parameters for the two new structures further highlights the large variation at dinucleotide and trinucleotide levels.

## Methods

### Crystal structure dataset selection

All X-ray crystal structures of the nucleosome core particle with a resolution better than 3.0 Å, released on or before 1*^st ^*November, 2009, were downloaded from the Protein Data Bank [48]. The dataset comprises of twenty-nine structures, which correspond to only six unique sequences and have been labelled with numbers 1 to 6 in our analysis (Table 1).

Sequences 1 and 2 had only one corresponding structure, namely 1KX4 [15] and 2NZD[31] respectively, sequences 3 and 6 had two corresponding structures, namely 1U35 [49] and 2F8N [31], and 1KX5 [15] and 2PYO [50] respectively, sequence 4 had three corresponding structures, namely 1AOI [51], 1KX3 [15] and 2CV5 [27], while the remaining twenty structures (1EQZ [52], 1F66 [53], 1M18, 1M19, 1M1A [54], 1P34, 1P3A, 1P3B, 1P3F, 1P3G, 1P3I, 1P3K, 1P3L, 1P3M, 1P3O, 1P3P [55], 1S32 [56], 1ZLA [57], 2NQB [31], 3C1B [58]) correspond to sequence 5.

Figure S15 in additional file 1 shows superhelical location (abbreviated as SHL) 0, which denotes the position of the pseudo two-fold axis of symmetry for the superhelix. At SHL 0, the major groove of the DNA faces the histone octamer. Before we proceed further, a few words about nomenclature are in order. As shown in figure S15, additional file 1 we will denote positions on the nucleosomal DNA sequence in terms of their displacement from SHL 0. The basepairs separated by factors of 10 from SHL 0 would be denoted by SHL ±i, where i is the relevant multiple of 10. The negative sign indicates a basepair on the 5'-side of SHL 0 along strand 1, while the positive sign indicates a basepair on the 3'-side of SHL 0 along strand 1. The basepairs between the integral super helical locations would be denoted by appropriate fractions. Thus, -4.4 would indicate a position four basepairs away from SHL 4, towards the 5'-end along strand 1, while 4.4 would indicate a position four basepairs away from SHL 4, towards the 3'-end along strand 1.

Sequence 4 is the 146-basepair long α satellite DNA. The 146-basepair long sequence 5 differs from it in two locations, with G:C and C:G basepairs replacing the T:A and A:T basepairs at positions -0.3 and 0.4 respectively. In sequence 6, the T:A and A:T basepairs at positions -5.2 and 5.3 in sequence 4 (the 146-basepair long α satellite DNA) are replaced by A:T and T:A basepairs respectively. In addition, a G:C basepair is present between positions -0.1 and -0.2 of sequence 4 to make it a 147-basepair long DNA. Finally, the T:A basepair at position 0.2 of sequence 4 is replaced by a C:G basepair to maintain basepair complementarity. Sequences 2 and 3 are 145-basepair long, with the T:A basepair at position 0.2 of sequence 4 missing. Further, the T:A and A:T basepairs at positions -5.2 and 5.3 in sequence 4 are replaced by A:T and T:A basepairs in case of sequence 2, and by G:C and C:G basepairs in case of sequence 3. 146-basepair long sequence 1 is very distinct from all the others, with differences at thirty-six positions in comparison to sequence 4.

In case of the structures corresponding to the 145-basepair long sequences 2 and 3, as well as the 147-basepair long sequence 6, SHL 0 passes through the central basepair, effectively dividing the structure into two equal halves. In case of the 146-basepair long sequences corresponding to sequences 1, 4 and 5, SHL 0 passes through the 73*^rd ^*basepair, counting from the 5'-end of strand 1. Thus SHL 0 divides the structure into two unequal halves, a shorter half of 72-basepairs on the 5'-side along strand 1, and a longer half of 73-basepairs on the 3'-side along strand 1.

All the structures corresponding to sequences 1 (PDB id 1KX4), 2 (PDB id 2NZD), 4 (PDB id's 1AOI, 1KX3 and 2CV5) and 6 (PDB id's 1KX5 and 2PYO), as well as one structure corresponding to sequence 3 (PDB id 2F8N) and two structures corresponding to sequence 5 (PDB id's 1EQZ and 2NQB) comprise of wild-type histones, though the histone sequence might vary at a few amino acid positions depending on the organism from which it was derived. None of these structures have any additional ligands attached to the DNA or the protein. The second structure corresponding to sequence 3 (PDB id 1U35) contains a histone variant macroH2A. The remaining eighteen structures corresponding to sequence 5 display a variety of histone mutations and binding by different ligands: mutations in histones H3 and H4 (PDB id's 1P34, 1P3A, 1P3B, 1P3F, 1P3G, 1P3I, 1P3K, 1P3L, 1P3M, 1P3O, 1P3P, 3C1B), mutation in H2A to give H2A.Z (PDB id 1F66), presence of a linker joining the two DNA disks (PDB id 1S32), presence of groove binding ligands (PDB id's 1M18, 1M19, 1M1A) and presence of an antigen bound to DNA (PDB id 1ZLA).

There are two structures of the nucleosome core particle (NCP) from Drosophila melanogaster (PDB id's 2NQB and 2PYO), and one structure each of the NCP from chicken (PDB id 1EQZ), mouse (PDB id 1U35) and human (PDB id 2NZD). All the other structures are of the NCP from Xenopus laevis.

### Classification of dinucleotide steps depending on their orientation with respect to the histone octamer

For each integer i, (-7 ≤ i ≤ 7), SHL i denotes the centre of the region where the DNA major groove faces the histone octamer. On the other hand, each half-integer value i.5 denotes the centre of the region where the DNA minor groove faces the histone octamer. Hence, for each SHL i, the four dinucleotide steps incorporating the five basepairs at positions (i - 1) + 0.8, (i - 1) + 0.9, i, i + 0.1 and i + 0.2 have been classified as belonging to the region where the major groove faces the histone octamer. Similarly the four dinucleotide steps incorporating the five basepairs at positions i + 0.3, i + 0.4, i + 0.5, i + 0.6 and i + 0.7 have been classified as belonging to the region where the minor groove faces the histone octamer. The steps intermediate between these two regions, i. e. incorporating basepairs i + 0.2 and i + 0.3, and i + 0.7 and i + 0.8, have been classified as belonging to the region where the DNA backbone faces the histone octamer.

### Calculation and classification of backbone torsion angles

Backbone torsion angles *α*, *β*, *γ*, *δ*, *ϵ*, *ζ *; the glycosidic torsion angle *χ *and the pseudo rotation angle P [59] were calculated using the NUPARM program [60-62]. Backbone torsion angles for a basepaired dinucleotide step, i.e., across the phosphodiester bond, were clustered and analysed. *ϵ*: C4*'_n_*-C3*'_n_*-O3*'_n_*-P*_n_*, ζ: C3*'*_*n*_-O3*'*_*n*_-P_*n*+1_-O5*'*_*n*+1_, *α*: O3*'_n_*-P_*n*+1_-O5*'*_*n*+1_-C5*'*_*n*+1 _and *γ*: O5*'*_*n*+1_-C5*'*_*n*+1_-C4*'*_*n*+1_-C3*'*_*n*+1 _are classified into seven states as per the algorithm proposed by Dixit et al. [63]. Since *ϵ *and ζ assume two related conformations, *ϵ*, *ζ *= t, g^− ^being the canonical conformation, known as BI, and *ϵ*, *ζ *= g^−^, t being the non-canonical conformation, known as BII, a value of *ϵ *- *ζ *≤ 0 has been classified as the BI conformation, and a value of *ϵ *- *ζ *> 0 has been classified as the BII conformation.

### Evaluation of basepair and dinucleotide step parameters

The structural parameters of the duplexes i.e. the basepair parameters buckle, propeller twist and open angle, the dinucleotide step parameters tilt, roll, twist, shift, slide, rise and Z*_p_*, and the bending angles between all dinucleotide steps were calculated using the NUPARM program [60-62].

The basepair parameters buckle, propeller twist and opening angle describe relative rotation between paired bases about their common x, y and z-axes respectively. All these parameters essentially quantify the intrinsic or induced basepair non-planarity.

The dinucleotide step parameters tilt, roll and twist measure the relative rotational motion between adjacent basepairs, while shift, slide and rise measure relative translational motion between adjacent basepairs along the local doublet x, y and z-directions respectively. The parameter Z*_p _*[28] is defined as the mean z-coordinate of the backbone phosphate atoms of the basepair with respect to the basepair dimer reference frame.

A local helix axis corresponding to each dinucleotide step is defined as the vector pointing in the direction of the cross-product of the differences of the x and y-vectors of the constituent basepair planes. The angle between two local helix axes vectors corresponding to overlapping dinucleotide steps is classified as the successive bending angle between those two steps.

### Calculation of minor groove width

For the fibre model of a N-basepair DNA, if the phosphates in strand 1 are numbered from 2 to N in the 5'-3*' *direction, and the phosphates in strand 2 are numbered from 1*' *to (N-1)*' *in the 3*'*-5*' *direction, the minimum interstrand phosphate-phosphate (P..P) separation on the minor groove side is between P_i+4 _and P_i_*'*, and spans 2 dinucleotide steps or 3 basepairs, and is in that sense, a trinucleotide parameter. We have classified the minimum P..P separation on the minor groove side as the minor groove width.

### Calculation of angle between successive, non-overlapping tetranucleotide and pentanucleotide fragments

A global helix axis for a given fragment of DNA has been defined as the best-fit line fitted to the backbone C_1_*' *atoms of all the nucleotides constituting that fragment. The angles between global helix axes corresponding to successive, non-overlapping tetranucleotide and pentanucleotide fragments were calculated. The angles were assigned the sign of their dot product with the average of vectors in the x-directions of the two central basepairs.

### Calculation of parameters quantifying curvature

The calculation of the radius of curvature using a least square circle fit method, the ratio of end-to-end distance to the contour length (d/l*_local _*or d/I*_max_*) and the three moments of inertia I*_max_*, I*_mid _*and I*_min _*were done as described previously in [64]. The radius of curvature (ROC) is calculated by fitting a circle to the basepair centres of the DNA molecules. Smaller the radius of this circle, the more curved is the DNA. However, the quality of the fit to a circle is affected by distortions at the local level in the duplex i.e. the successive bending angles. Thus the presence of several trinucleotides that are distorted, even to a small degree, would lead to a poor circle fit and consequently an inaccurate value of ROC. After a careful consideration of the RMSD values for a circle fit and a line fit for all the nucleosome fragments under consideration, only those fragments for which the RMSD for a circle fit is ≤ 1.5 Å and the RMSD for a line fit is ≥ 10.8 Å have been included, for the calculation of mean and standard deviation values for all parameters quantifying curvature. Most of the fragments for all the structures in our dataset were observed to satisfy these criteria, with the minimum number of selected fragments being 99 out of 109 for the structure with PDB id 1F66.

### Calculation of autocorrelation and crosscorrelation values

The calculation of autocorrelation values for parameters of the same structure, and cross-correlation values for parameters of different structures were carried out using MATLAB. For calculation of crosscorrelation values, the parameter sets for 146 and 147-basepair sequences were truncated at the ends, so that the parameter sets for 145, 146 and 147-basepair sequences were of equal size. Care was taken to ensure that at zero lag, the parameter sets were aligned with respect to values corresponding to SHL 0 position.

All the analysis has been carried out excluding terminal basepairs to eliminate end effects. All the plots were generated using MATLAB.

## Authors' contributions

AM and MB designed the project. AM carried out the analysis and drafted the manuscript. The project was supervised by MB. Both the authors read and approved the manuscript.

## Supplementary Material

Additional file 1 **Structural variation at different length scales in nucleosomal DNA**. This file contains mean and standard deviation values for three of the dinucleotide parameters, the trajectories for dinucleotide, trinucleotide, octanucleotide and decanucleotide parameters for six representative structures, and the autocorrelation and cross-correlation values between these parameters for the same six structures. Click here for file
