# Supplementary material for: An ensemble of B-DNA dinucleotide geometries lead to characteristic nucleosomal DNA structure and provide plasticity required for gene expression
Source: BMC Struct Biol. 2011 Jan 5;11:1. doi: 10.1186/1472-6807-11-1 (PMC3031206; doi:10.1186/1472-6807-11-1)
Supplement: Additional file 1 — Structural variation at different length scales in nucleosomal DNA. This file contains mean and standard deviation values for three of the dinucleotide parameters, the trajectories for dinucleotide, trinucleotide, octanucleotide and decanucleotide parameters for six representative structures, and the autocorrelation and cross-correlation values between these parameters for the same six structures. [file 1472-6807-11-1-S1.PDF]

Structural variation at different length scales in nucleosomal DNA

Additional file 1

for the article

An ensemble of B-DNA dinucleotide geometries lead to characteristic nucleosomal DNA structure and provide plasticity required for gene expression

Arvind Marathe and Manju Bansal

Table S1: Mean and standard deviation values (given in parentheses, reported only for datasets with size ≥ 5) for tilt, for the ten dinucleotide steps, incorporating Watson-Crick basepairs

Step	Tilt values for dinucleotide steps with								
	Minor groove (Region I)			Backbone facing the histone octamer (Region II)			Major groove (Region III)		
	BI/BI	BI/BII	BII/BII	BI/BI	BI/BII	BII/BII	BI/BI	BI/BII	BII/BII
	-0.8	-0.7	3.1	-1.2	-0.9	5.3	-0.9	-1.7	-2.7
AA/TT	(2.5)	(3.6)	(3.9)	(3.4)	(4.3)	NA	(2.0)	(2.9)	NA
	241	67	8	122	21	1	289	55	1
	-0.7	0.6	1.0	-3.2	-9.4	NA	-2.1	-3.8	NA
AG/CT	(2.9)	(2.9)	(2.8)	(2.7)	NA		(3.4)	(3.3)	
	104	119	47	29	2	0	139	38	0
	-1.8	-2.4	-0.5	-2.2	-2.3	-3.1	-2.6	-3.7	-1.5
GA/TC	(2.7)	(3.5)	NA	(3.0)	(3.4)	NA	(2.7)	(3.1)	NA
	82	117	2	73	31	3	153	54	2
	-0.8	0.9	-1.7	-3.6	-3.0	2.1	-3.9	-3.4	NA
GG/CC	(2.1)	(3.6)	(2.9)	(3.7)	(4.8)	NA	(2.8)	(3.9)	
	71	150	28	42	16	1	75	13	0
	0.6	-0.5	NA	0.4	-1.5	NA	-0.4	-1.7	NA
AC/GT	NA	NA		(2.0)	NA		(2.9)	(5.3)	
	1	4	0	59	1	0	271	20	0
	0.1	-0.4	NA	1.3	2.5	NA	-0.9	0.6	NA
AT/AT	(2.1)	(2.7)		(2.0)	NA		(2.2)	(1.5)	
	109	5	0	110	4	0	139	6	0
	-1.8	-0.4	1.4	0.2	-1.9	NA	-2.8	-0.8	NA
GC/GC	(4.6)	(3.2)	(3.1)	(2.7)	(2.4)		(3.4)	NA	
	32	102	12	29	28	0	26	1	0
	-1.7	-0.8	2.6	0.2	-1.0	2.3	0.8	0.6	-0.9
CA/TG	(1.7)	(2.7)	(2.9)	(3.6)	(4.0)	(3.2)	(2.5)	(3.5)	(2.5)
	54	65	120	72	82	54	158	99	51
	-1.4	1.2	0.4	NA	NA	NA	-1.0	4.3	NA
CG/CG	NA	(2.1)	(2.1)				NA	NA	
	3	35	10	0	0	0	1	1	0
	0.3	-0.9	-7.9	-0.1	-1.5	-2.7	-0.5	3.6	-1.0
TA/TA	(2.2)	NA	NA	(1.6)	(2.2)	NA	(3.2)	(2.6)	NA
	33	2	1	15	16	1	82	29	1
	-0.8	-0.2	-1.5	-0.7	-1.5	2.0	-1.1	-1.1	-1.0
Overall	(2.6)	(3.5)	(3.3)	(3.3)	(3.9)	(3.5)	(2.9)	(4.1)	NA
	730	666	228	551	201	60	1333	316	55

For each dinucleotide step, the 3rd row lists the size of the population for the respective dataset. Based on the side of the DNA facing the histone octamer (minor groove, backbone or major groove), the dinucleotide steps have been divided into three groups. The population in each group has been further sub-divided depending on whether the backbone conformation was BI in both strands, or a mixture of BI and BII, or BII in both strands. Please refer 'Methods' section in the main article for details of the classification scheme.

Table S2: Mean and standard deviation values (given in parentheses, reported only for datasets with size ≥ 5) for shift, for the ten dinucleotide steps, incorporating Watson-Crick basepairs

Step	Shift values for dinucleotide steps with Minor groove (Region I)			Backbone facing the histone octamer (Region II)			Major groove (Region III)		
	BI/BI	BI/BII	BII/BII	BI/BI	BI/BII	BII/BII	BI/BI	BI/BII	BII/BII
AA/TT	0.2	0.2	0.5	-0.1	-0.4	0.7	-0.0	-0.3	0.1
	(0.3)	(0.5)	(0.2)	(0.3)	(0.4)	NA	(0.3)	(0.4)	NA
	241	67	8	122	21	1	289	55	1
AG/CT	0.3	0.5	0.3	-0.0	-1.7	NA	0.0	-0.5	NA
	(0.5)	(0.9)	(0.4)	(0.3)	NA		(0.7)	(0.8)	
	104	119	47	29	2	0	139	38	0
GA/TC	-0.3	-0.8	-0.6	-0.3	-0.7	-0.9	-0.3	-0.5	-0.1
	(0.3)	(0.6)	NA	(0.6)	(0.6)	NA	(0.3)	(0.6)	NA
	82	117	2	73	31	3	153	54	2
GG/CC	0.1	0.5	-0.2	-0.2	0.1	0.9	-0.3	-0.2	NA
	(0.4)	(0.8)	(0.5)	(0.5)	(0.8)	NA	(0.3)	(0.7)	
	71	150	28	42	16	1	75	13	0
AC/GT	0.2	0.7	NA	0.3	0.8	NA	0.2	-0.4	NA
	NA	NA		(0.3)	NA		(0.4)	(0.7)	
	1	4	0	59	1	0	271	20	0
AT/AT	0.0	0.1	NA	0.1	0.1	NA	-0.1	0.1	NA
	(0.3)	(0.5)		(0.4)	NA		(0.4)	(0.5)	
	109	5	0	110	4	0	139	6	0
GC/GC	-0.1	-0.4	-0.1	-0.2	-0.7	NA	0.1	0.6	NA
	(0.5)	(0.9)	(0.5)	(0.3)	(0.5)		(0.3)	NA	
	32	102	12	29	28	0	26	1	0
CA/TG	-0.3	-0.4	0.2	0.1	-0.1	0.3	-0.2	0.0	-0.1
	(0.4)	(0.6)	(0.5)	(0.6)	(0.7)	(0.4)	(0.3)	(0.6)	(0.3)
	54	65	120	72	82	54	158	99	51
CG/CG	-0.2	0.6	0.3	NA	NA	NA	-0.0	0.3	NA
	NA	(0.6)	(0.5)				NA	NA	
	3	35	10	0	0	0	1	1	0
TA/TA	-0.0	-0.1	0.2	0.1	-0.2	-0.4	0.0	0.8	0.7
	(0.4)	NA	NA	(0.3)	(0.5)	NA	(0.3)	(0.6)	NA
	33	2	1	15	16	1	82	29	1
Overall	0.1	0.0	0.1	-0.0	-0.3	0.2	-0.0	-0.1	-0.1
	(0.4)	(0.9)	(0.5)	(0.5)	(0.7)	(0.5)	(0.4)	(0.7)	(0.3)
	730	666	228	551	201	60	1333	316	55

The specifications are as described in the legend for table S1.

Table S3: Mean and standard deviation values (given in parentheses, reported only for datasets with size ≥ 5) for rise, for the ten dinucleotide steps, incorporating Watson-Crick basepairs

Step	Rise values for dinucleotide steps with: Minor groove (Region I)			Backbone facing the histone octamer (Region II)			Major groove (Region III)		
	BI/BI	BI/BII	BII/BII	BI/BI	BI/BII	BII/BII	BI/BI	BI/BII	BII/BII
AA/TT	3.3	3.3	3.3	3.2	3.2	3.5	3.3	3.3	3.3
	(0.1)	(0.2)	(0.1)	(0.1)	(0.2)	NA	(0.1)	(0.1)	NA
	241	67	8	122	21	1	289	55	1
AG/CT	3.3	3.3	3.4	3.3	3.3	NA	3.4	3.3	NA
	(0.2)	(0.1)	(0.1)	(0.2)	NA		(0.1)	(0.1)	
	104	119	47	29	2	0	139	38	0
GA/TC	3.2	3.3	3.2	3.3	3.4	3.4	3.3	3.2	3.2
	(0.1)	(0.2)	NA	(0.2)	(0.2)	NA	(0.1)	(0.2)	NA
	82	117	2	73	31	3	153	54	2
GG/CC	3.3	3.4	3.9	3.3	3.3	3.3	3.3	3.2	NA
	(0.1)	(0.3)	(0.6)	(0.1)	(0.2)	NA	(0.1)	(0.1)	
	71	150	28	42	16	1	75	13	0
AC/GT	3.6	3.4	NA	3.3	3.6	NA	3.3	3.3	NA
	NA	NA		(0.1)	NA		(0.1)	(0.2)	
	1	4	0	59	1	0	271	20	0
AT/AT	3.2	3.0	NA	3.3	3.1	NA	3.3	3.2	NA
	(0.1)	(0.2)		(0.1)	NA		(0.1)	(0.1)	
	109	5	0	110	4	0	139	6	0
GC/GC	3.6	3.4	3.5	3.3	3.3	NA	3.3	3.4	NA
	(0.4)	(0.2)	(0.1)	(0.2)	(0.1)		(0.1)	NA	
	32	102	12	29	28	0	26	1	0
CA/TG	3.4	3.4	3.4	3.4	3.4	3.3	3.5	3.5	3.4
	(0.1)	(0.1)	(0.1)	(0.2)	(0.2)	(0.2)	(0.2)	(0.2)	(0.1)
	54	65	120	72	82	54	158	99	51
CG/CG	3.5	3.4	3.5	NA	NA	NA	3.0	3.4	NA
	NA	(0.1)	(0.1)				NA	NA	
	3	35	10	0	0	0	1	1	0
TA/TA	3.3	3.3	3.9	3.3	3.3	3.4	3.4	3.2	3.2
	(0.1)	NA	NA	(0.1)	(0.1)	NA	(0.2)	(0.1)	NA
	33	2	1	15	16	1	82	29	1
Overall	3.3	3.4	3.5	3.3	3.3	3.3	3.3	3.3	3.4
	(0.2)	(0.2)	(0.3)	(0.1)	(0.2)	(0.1)	(0.2)	(0.2)	(0.1)
	730	666	228	551	201	60	1333	316	55

The specifications are as described in the legend for table S1.

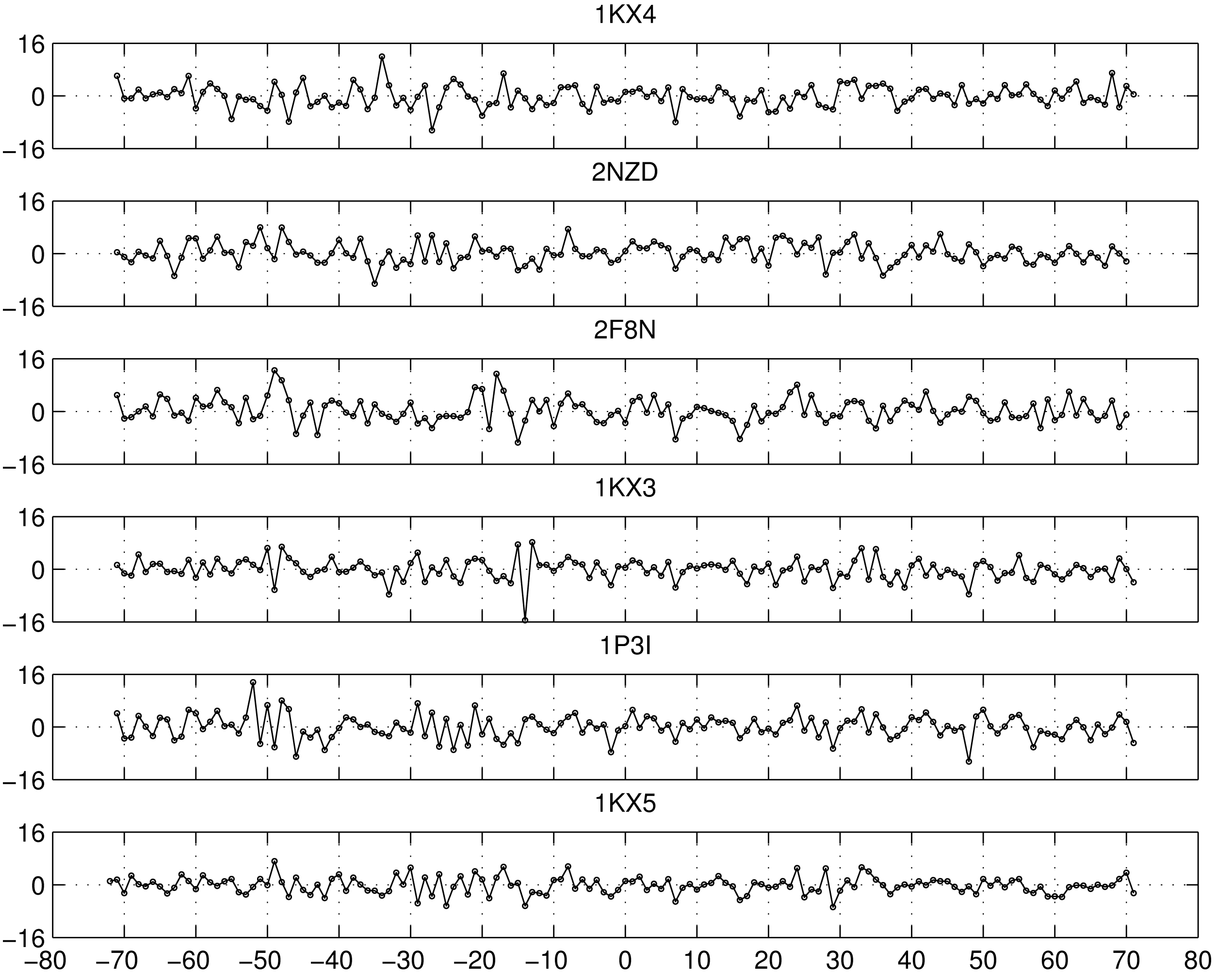

Figure S1: Tilt values for one representative structure corresponding to each of the six unique sequences. The PDB id's of the structures for which the values are shown are: 1KX4, 2NZD, 2F8N, 1KX3, 1P3I, 1KX5. Each value is shown corresponding to the first basepair of the dinucleotide.

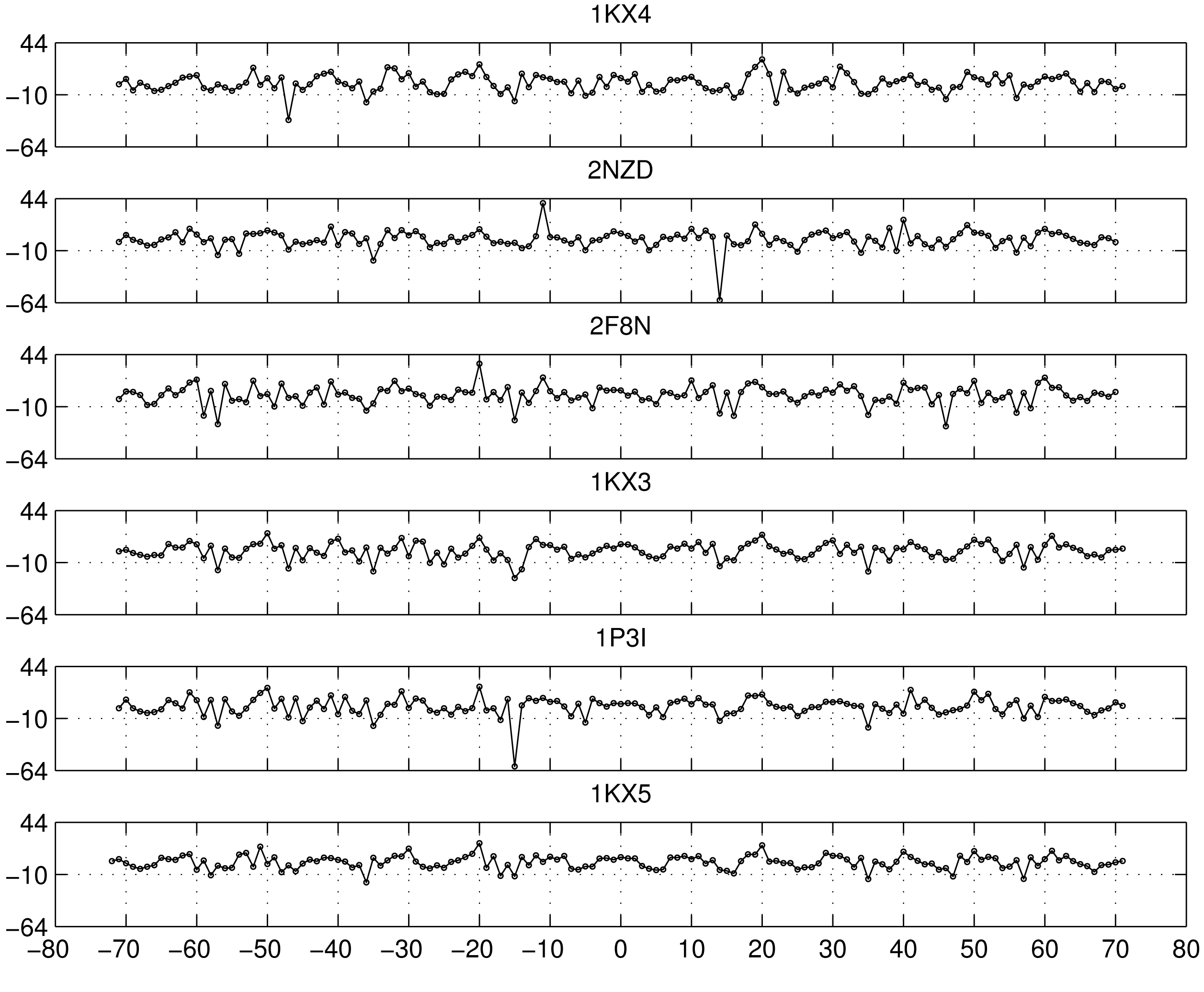

Figure S2: Roll values for one representative structure corresponding to each of the six unique sequences. The PDB id's of the structures for which the values are shown are: 1KX4, 2NZD, 2F8N, 1KX3, 1P3I, 1KX5. Each value is shown corresponding to the first basepair of the dinucleotide.

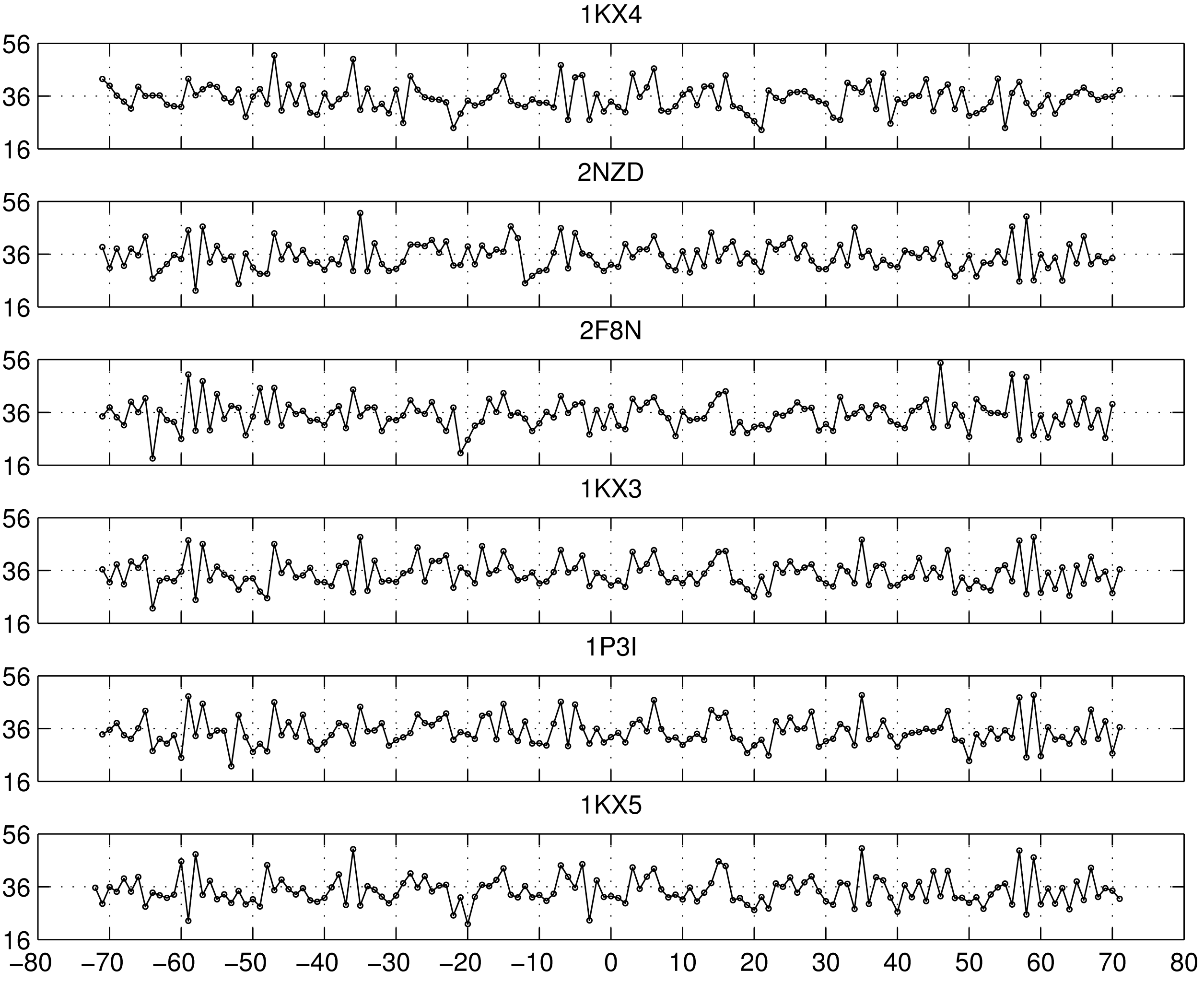

Figure S3: Twist values for one representative structure corresponding to each of the six unique sequences. The PDB id's of the structures for which the values are shown are: 1KX4, 2NZD, 2F8N, 1KX3, 1P3I, 1KX5. Each value is shown corresponding to the first basepair of the dinucleotide.

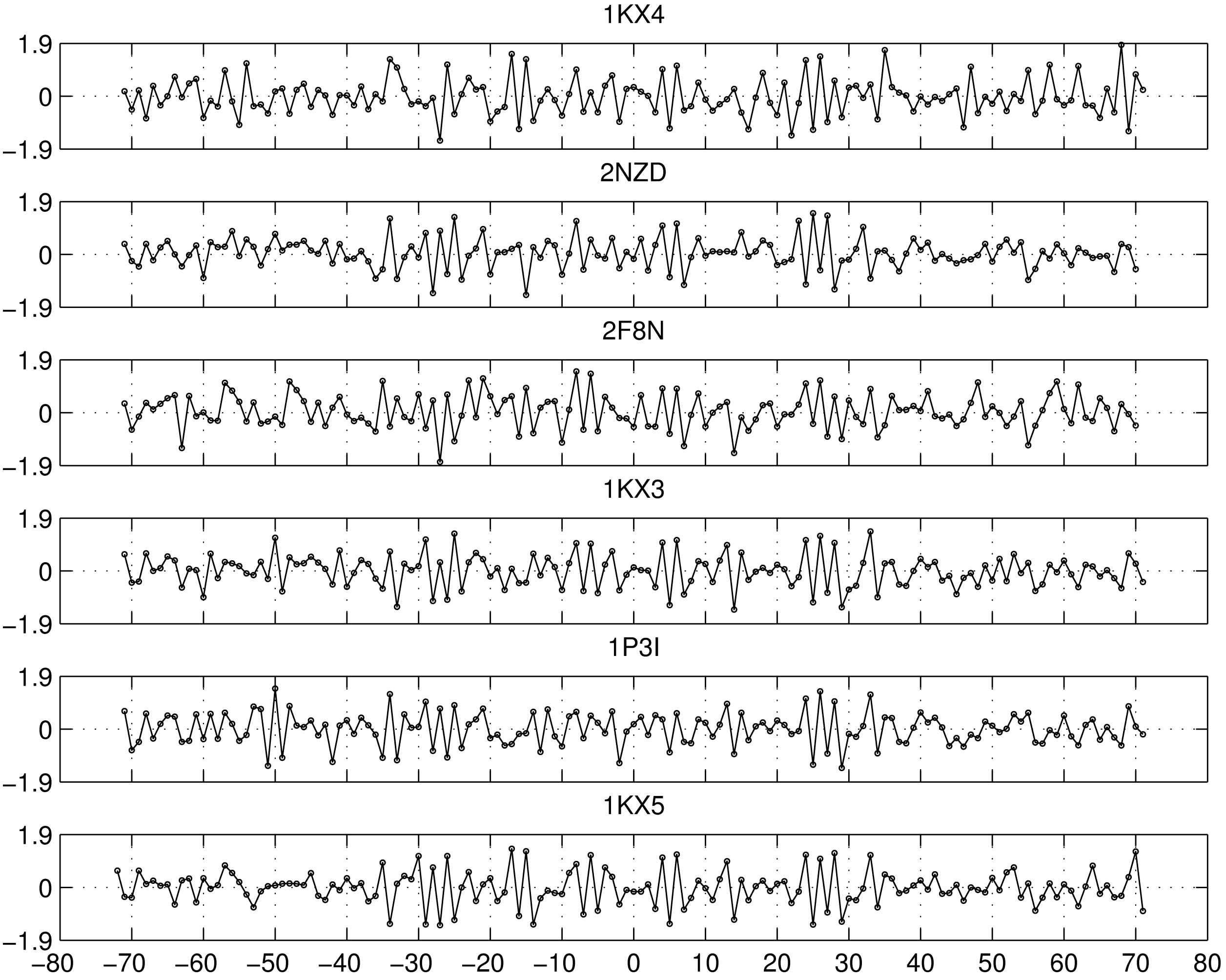

Figure S4: Shift values for one representative structure corresponding to each of the six unique sequences. The PDB id's of the structures for which the values are shown are: 1KX4, 2NZD, 2F8N, 1KX3, 1P3I, 1KX5. Each value is shown corresponding to the first basepair of the dinucleotide.

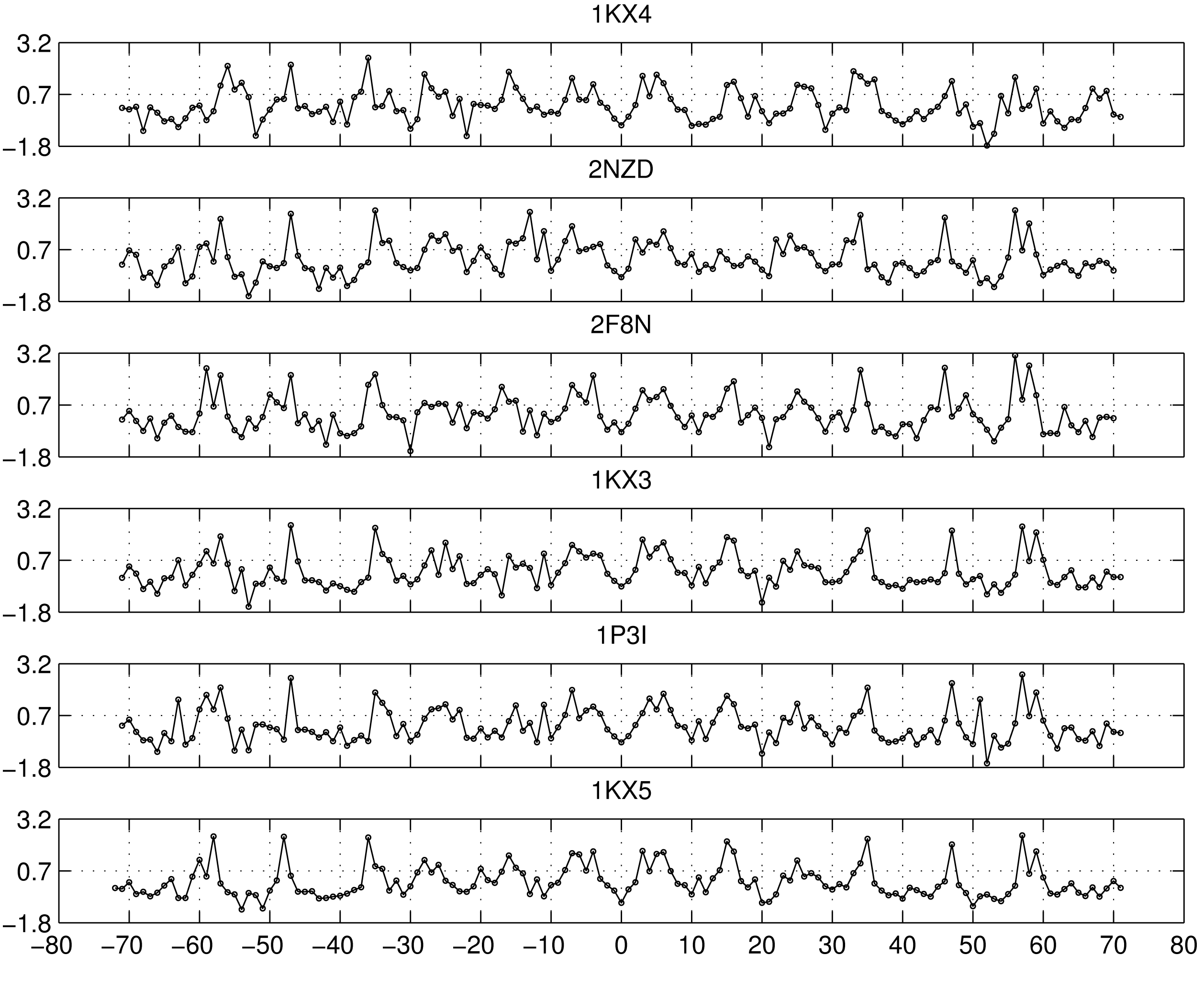

Figure S5: Slide values for one representative structure corresponding to each of the six unique sequences. The PDB id's of the structures for which the values are shown are: 1KX4, 2NZD, 2F8N, 1KX3, 1P3I, 1KX5. Each value is shown corresponding to the first basepair of the dinucleotide.

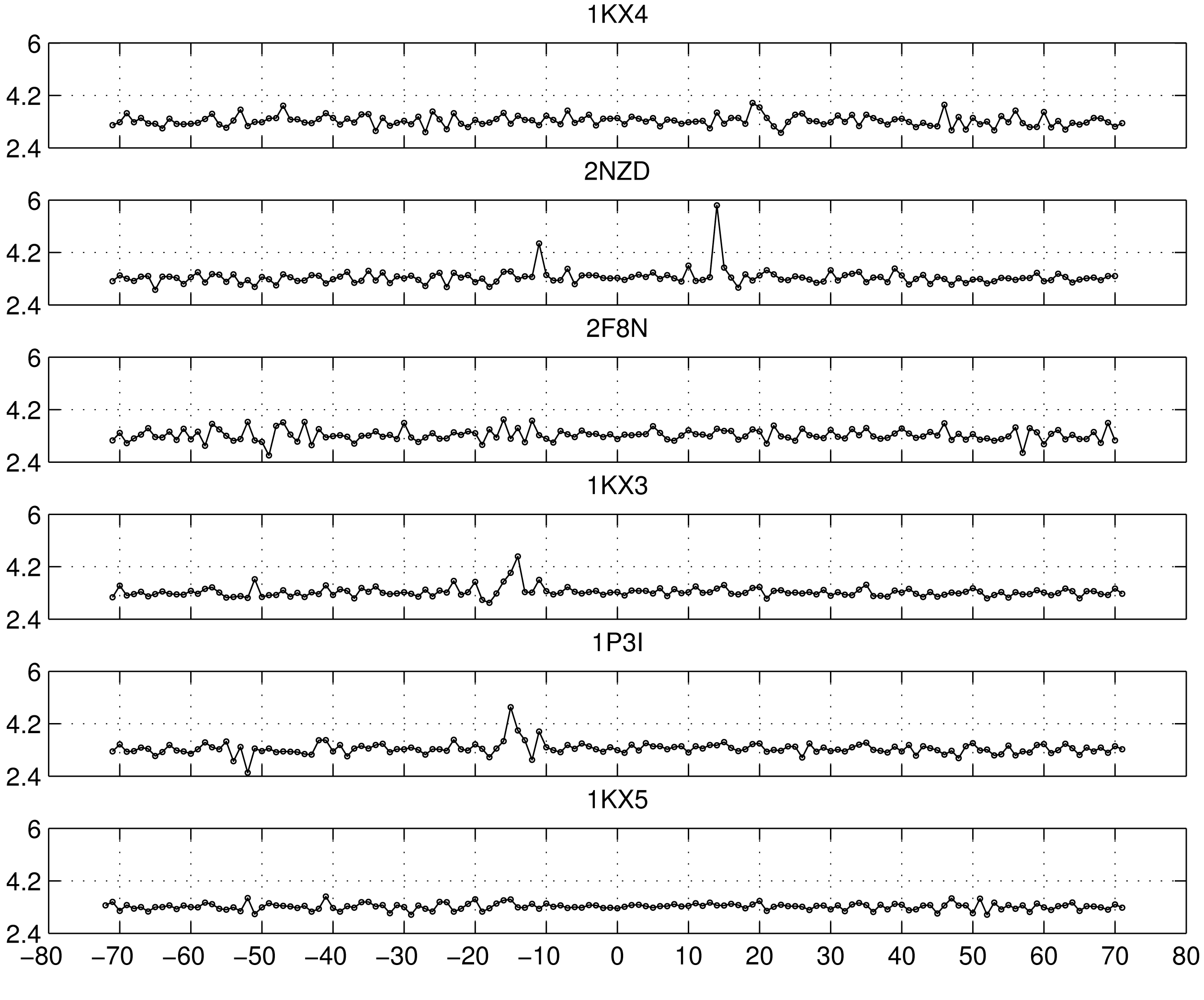

Figure S6: Rise values for one representative structure corresponding to each of the six unique sequences. The PDB id's of the structures for which the values are shown are: 1KX4, 2NZD, 2F8N, 1KX3, 1P3I, 1KX5. Each value is shown corresponding to the first basepair of the dinucleotide.

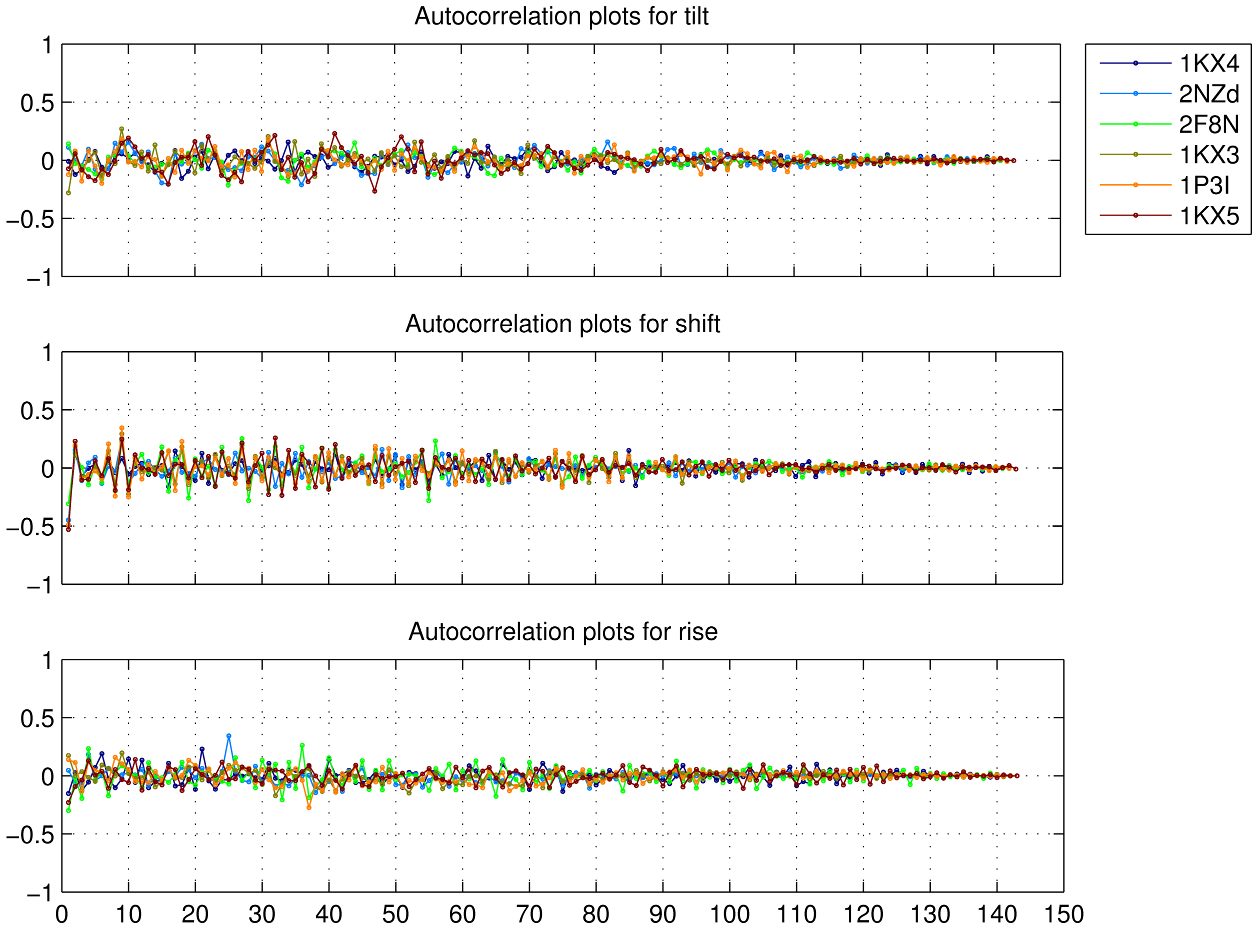

Figure S7: Autocorrelation values for the rotational parameter tilt, and the translational parameters shift and rise, for the six structures with PDB id's 1KX4, 2NZD, 2F8N, 1KX3, 1P3I and 1KX5, corresponding to sequences 1, 2, 3, 4, 5 and 6 respectively.

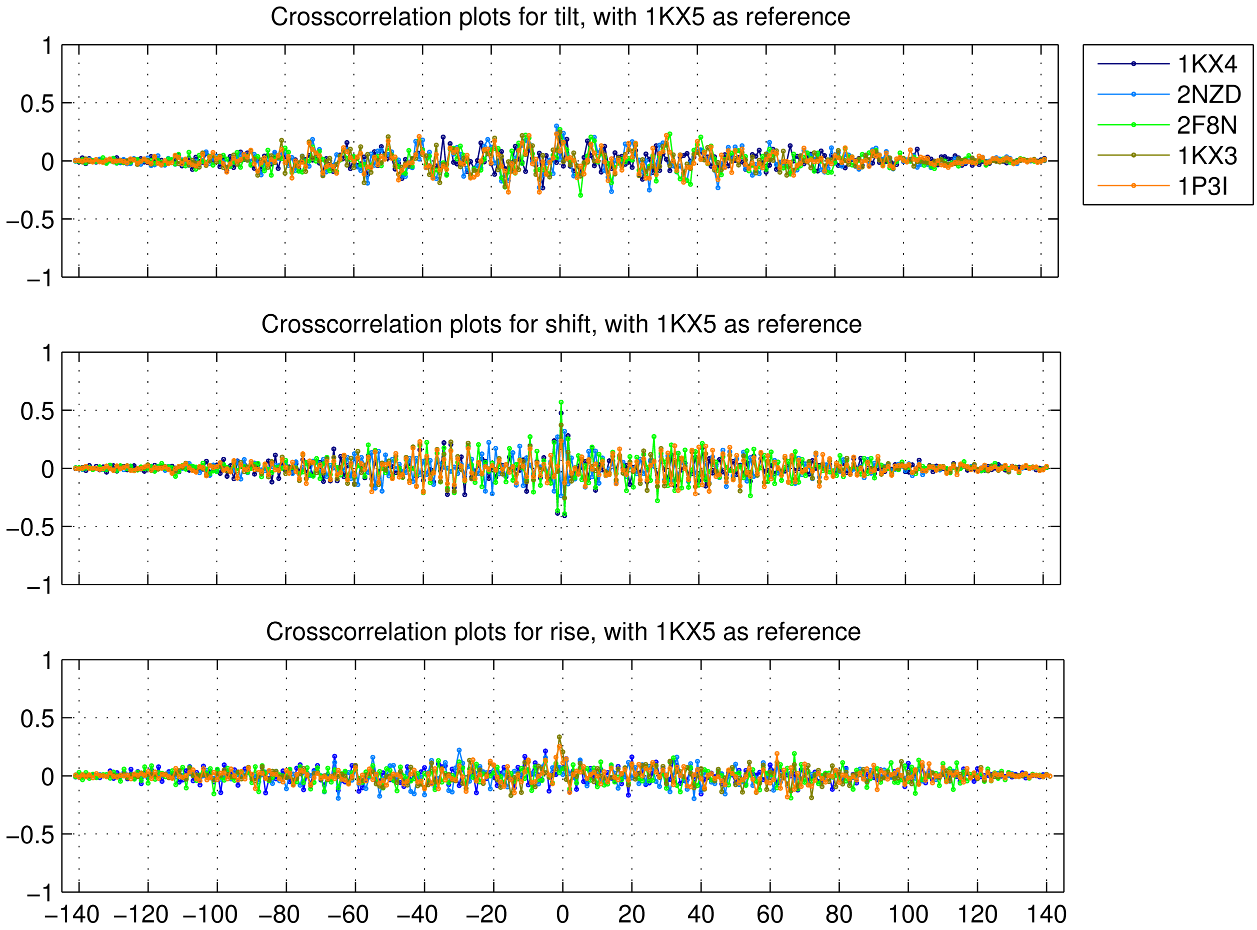

Figure S8: Crosscorrelation values for the rotational parameter tilt, and the translational parameters shift and rise, for the five structures with PDB id's 1KX4, 2NZD, 2F8N, 1KX3 and 1P3I, corresponding to sequences 1, 2, 3, 4 and 5, with respect to the corresponding parameters for the best resolved crystal structrure of the nucleosome with PDB id 1KX5, corresponding to sequence 6.

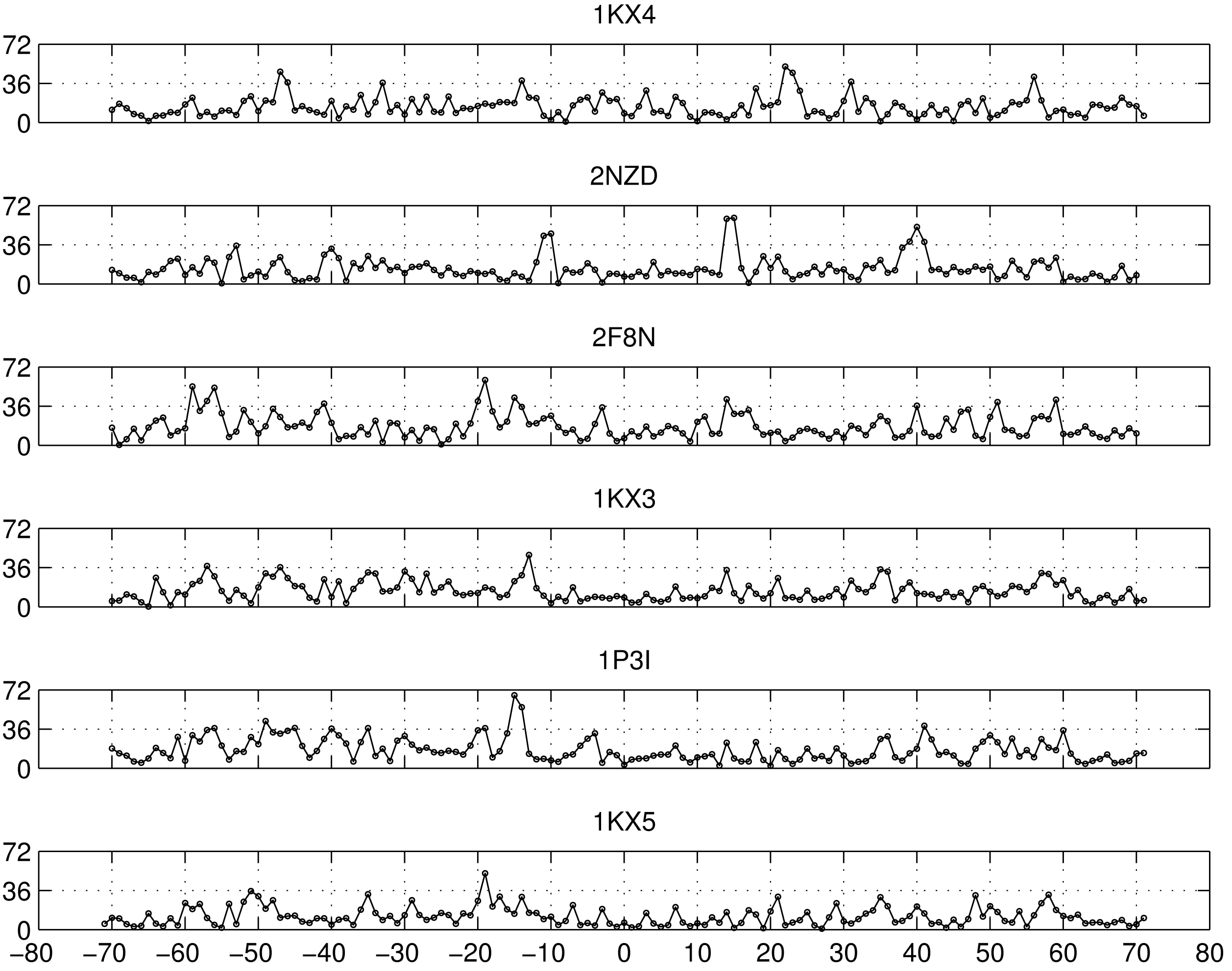

Figure S9: Successive bending angle values for one representative structure corresponding to each of the six unique sequences. The PDB id's of the structures for which the values are shown are: 1KX4, 2NZD, 2F8N, 1KX3, 1P3I, 1KX5. Each value is shown corresponding to the central basepair of the trinucleotide.

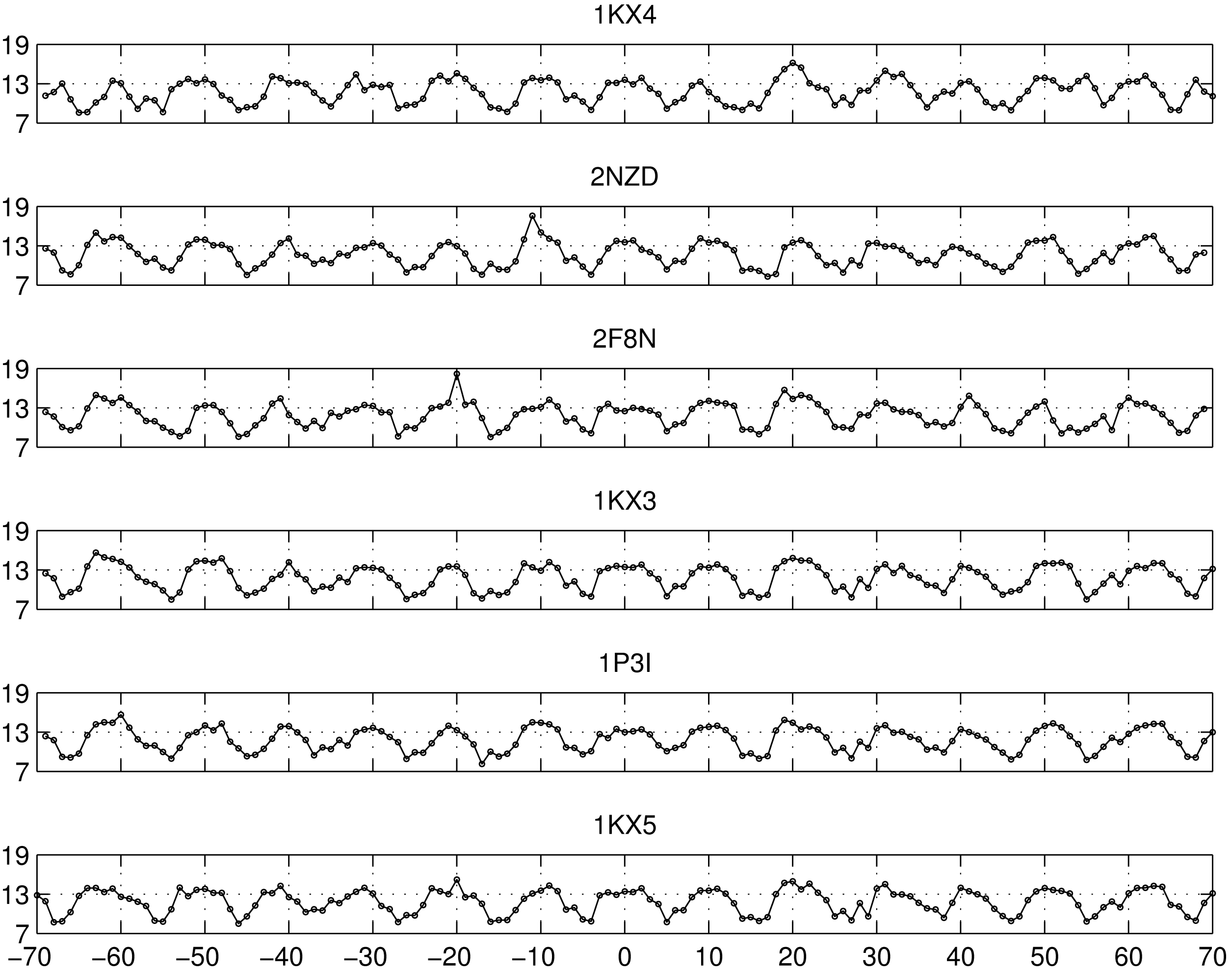

Figure S10: Minor groove width values for one representative structure corresponding to each of the six unique sequences. The PDB id's of the structures for which the values are shown are: 1KX4, 2NZD, 2F8N, 1KX3, 1P3I, 1KX5. Each value is shown corresponding to the central basepair of the trinucleotide.

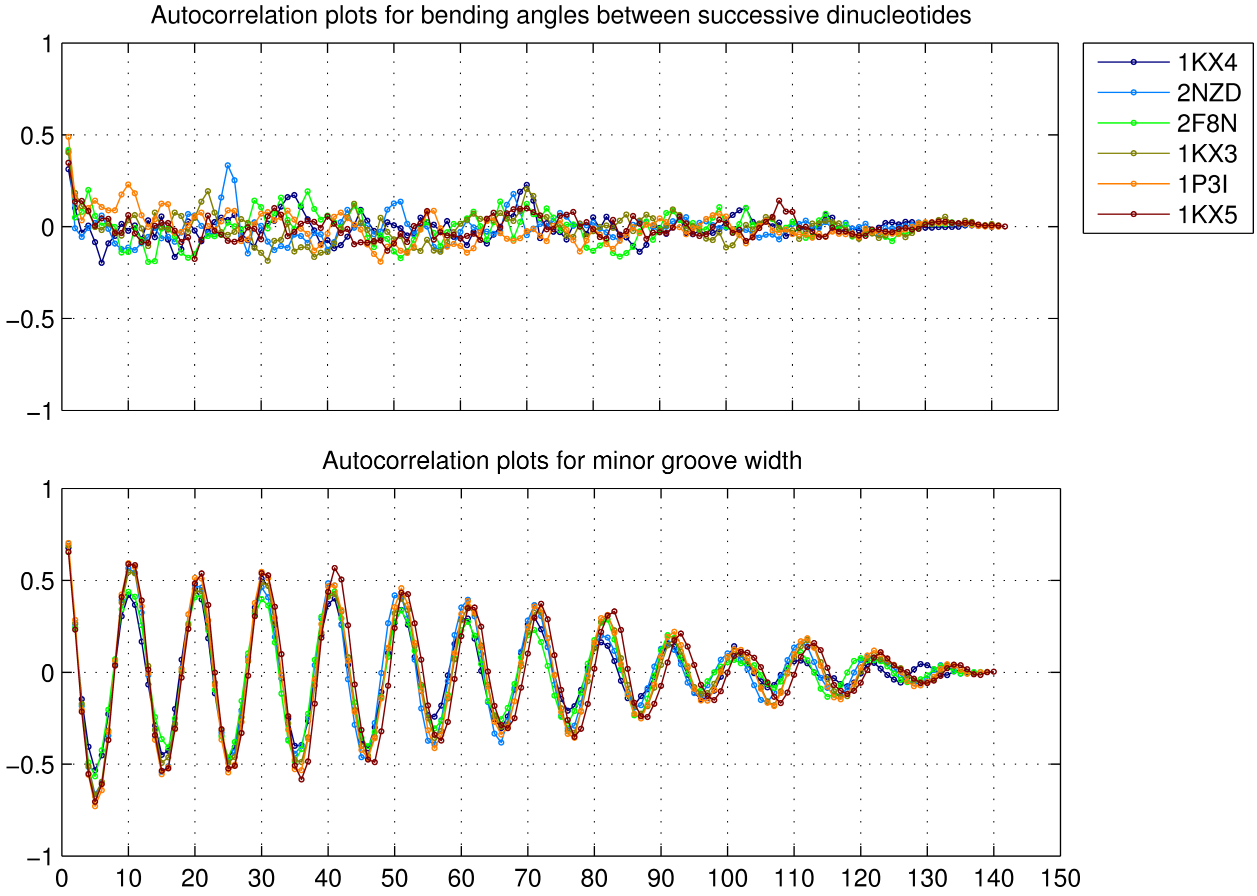

Figure S11: Autocorrelation values for the successive bending angles, and the minor groove width, for the six structures with PDB id's 1KX4, 2NZD, 2F8N, 1KX3, 1P3I and 1KX5, corresponding to sequences 1, 2, 3, 4, 5 and 6 respectively.

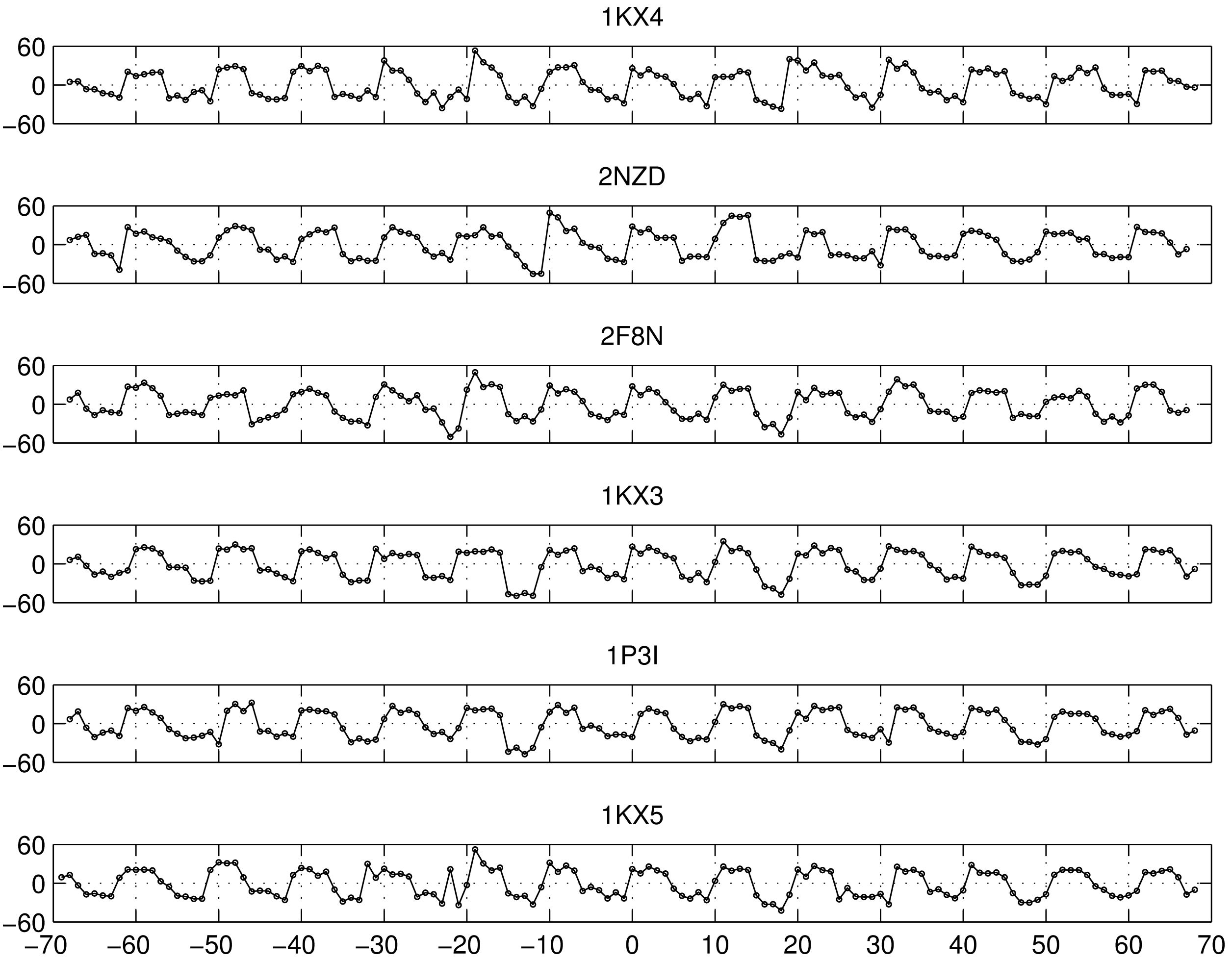

Figure S12: Angles between global helix axes fitted to backbone $C_1{}'$ atoms of successive, non-overlapping tetranucleotide fragments, for one representative structure corresponding to each of the six unique sequences. Each value is shown corresponding to the 4th position in the octanucleotide. The angles are assigned the sign of their dot product with the average of vectors in the x-directions of the two central basepairs. The structures for which the values are shown are: 1KX4, 2NZD, 2F8N, 1KX3, 1P3I, 1KX5.

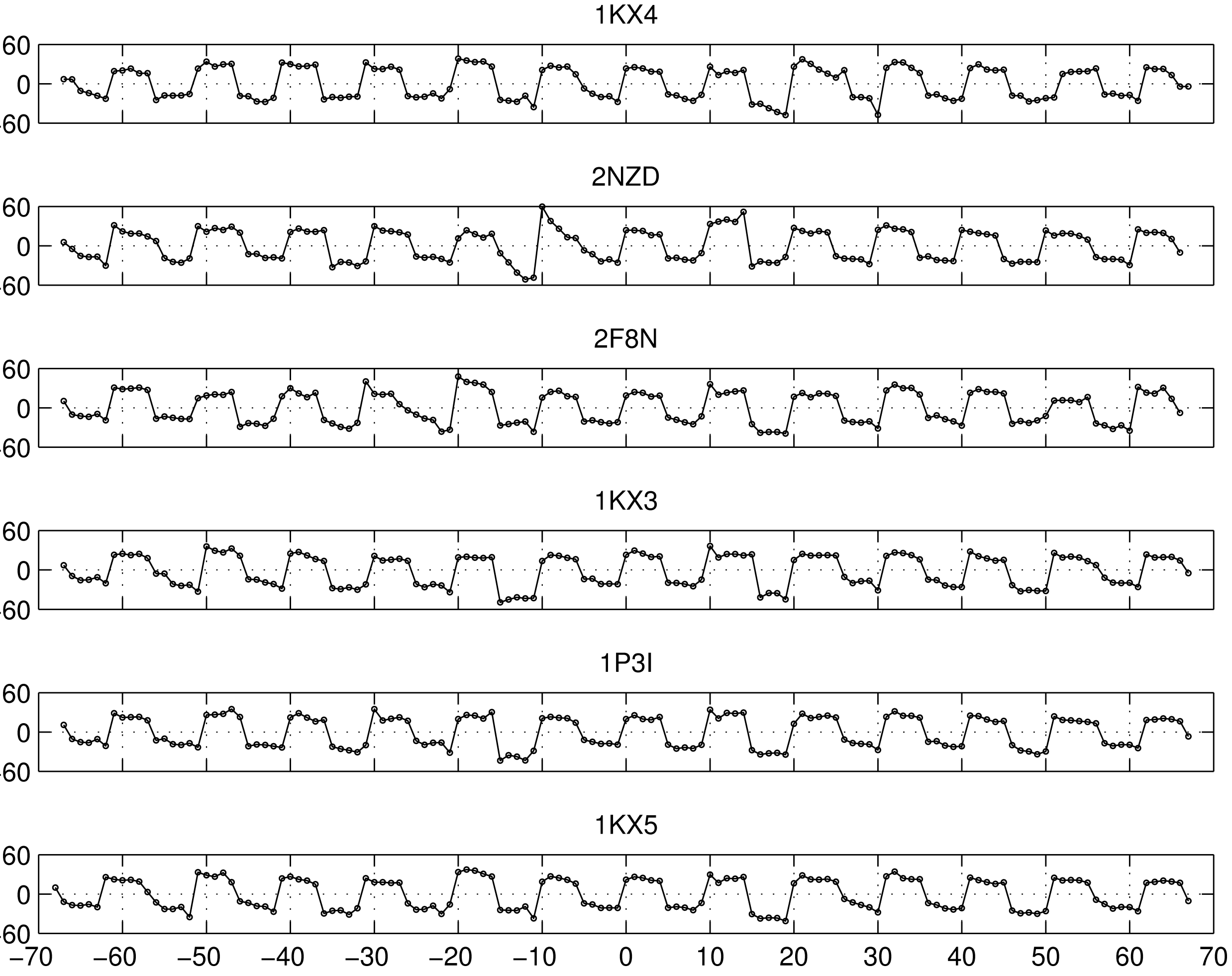

Figure S13: Angles between global helix axes fitted to backbone C_1' atoms of successive, non-overlapping pentanucleotide fragments, for one representative structure corresponding to each of the six unique sequences. Each value is shown corresponding to the 5th position in the decanucleotide. The angles are assigned the sign of their dot product with the average of vectors in the x-directions of the two central basepairs. The structures for which the values are shown are: 1KX4, 2NZD, 2F8N, 1KX3, 1P3I, 1KX5.

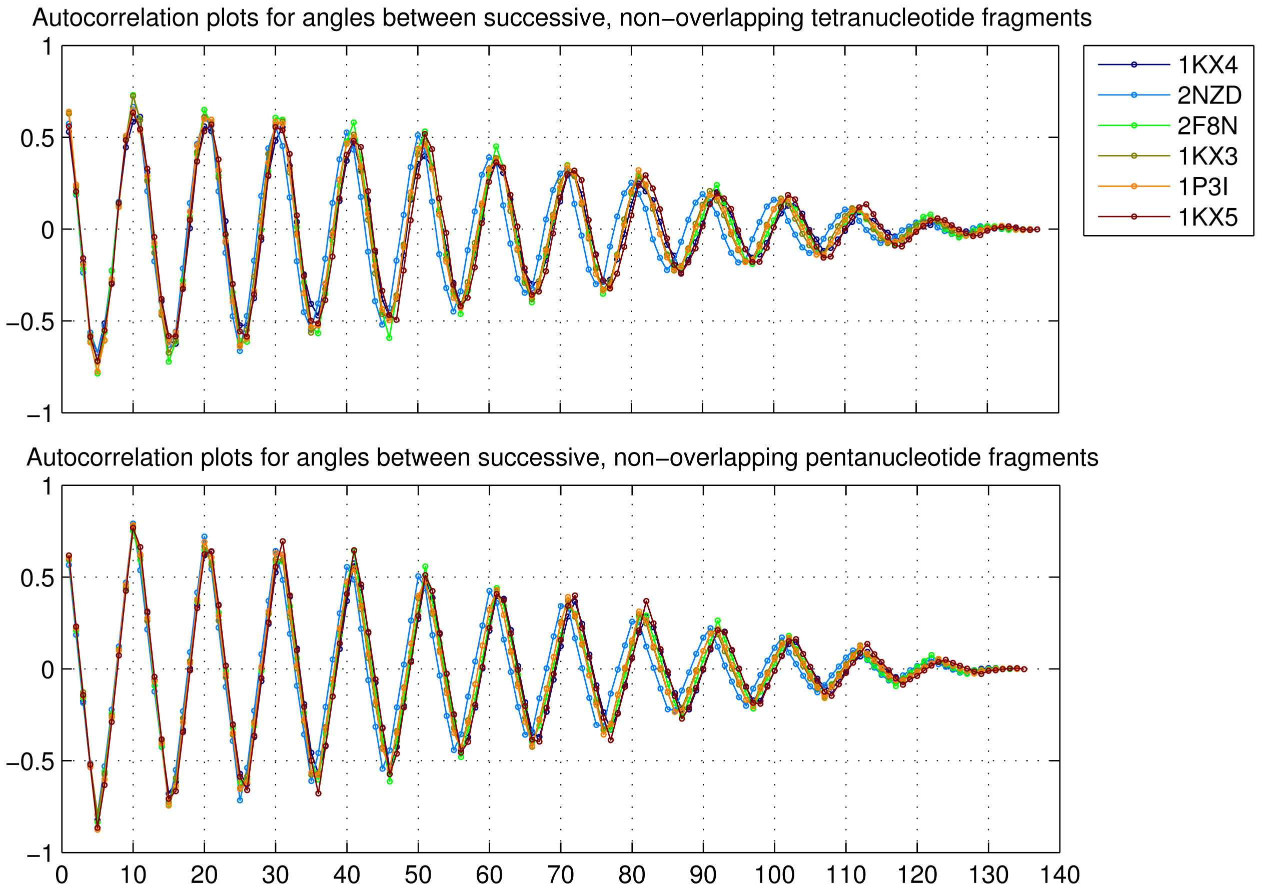

Figure S14: Autocorrelation values for the angles between global helix axes fitted to backbone C_1' atoms of successive, non-overlapping tetranucleotide fragments, and the angles between global helix axes fitted to backbone C_1' atoms of successive, non-overlapping pentanucleotide fragments, for the six structures with PDB id's 1KX4, 2NZD, 2F8N, 1KX3, 1P3I and 1KX5, corresponding to sequences 1, 2, 3, 4, 5 and 6 respectively. The rest of the specifications are as described in the captions for figures S12 and S13.

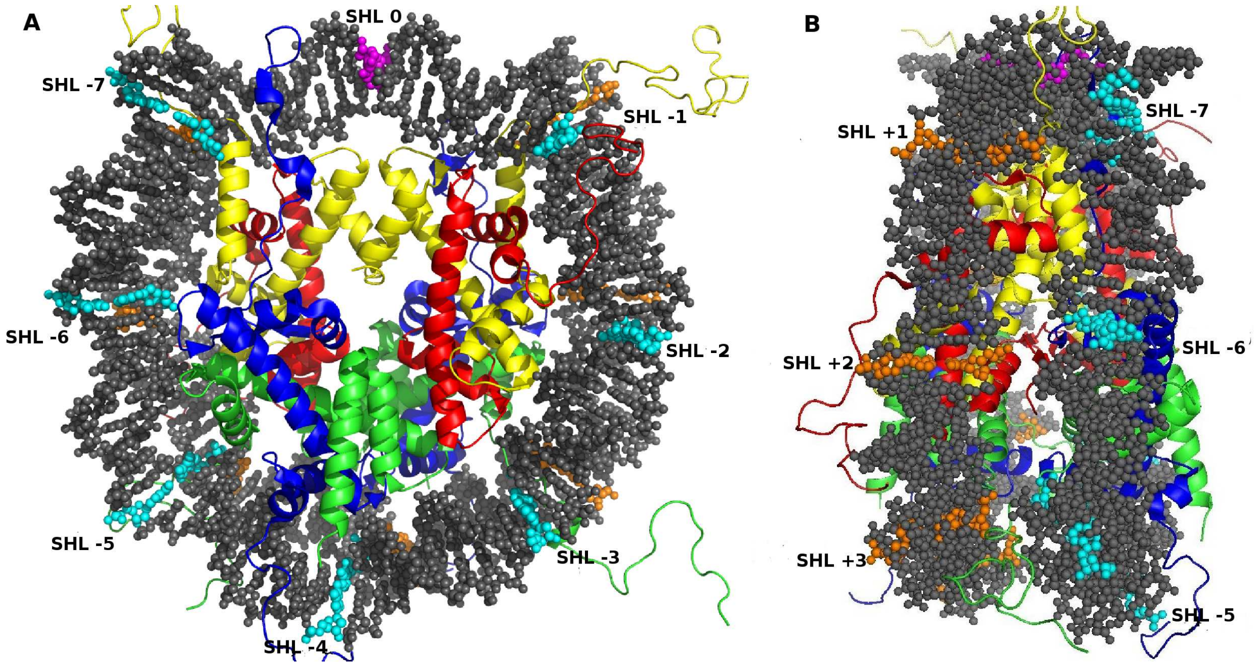

Figure S15: The structure of the nucleosome (PDB id 1KX5 [1]) showing (A) super helical locations (SHL) 0, -1, -2, -3, -4, -5, -6 and -7 (the front view) and (B) SHL -7, -6, -5 and +1, +2, +3 (the side view). For both subfigures, the colour code is as follows: Dark gray: Nucleosomal DNA, Magenta: SHL 0, Cyan: SHL -7 to -1, Orange: SHL +1 to +7, Yellow: Histone chains H3 and H3′, Red: Histone chains H4 and H4′, Blue: Histone chains H2A and H2A′, Green: Histone chains H2B and H2B′.

References

[1] Davey CA, Sargent DF, Luger K, Maeder AW, Richmond TJ: **Solvent mediated interactions in the structure of the nucleosome core particle at 1.9 Å resolution**. *J. Mol. Biol.* 2002, **319**:1097–1113.
